# Synthesis and Thermal Investigations of Eleven-Membered Ring Systems Containing One of the Heavier Group 14 Element Atoms Si, Ge, and Sn

**DOI:** 10.3390/molecules25020283

**Published:** 2020-01-10

**Authors:** Nadi Eleya, Clement Appiah, Enno Lork, Mathias Gogolin, Thorsten M. Gesing, Tim Stauch, Anne Staubitz

**Affiliations:** 1 Institute for Analytical and Organic Chemistry, University of Bremen, Leobener Straße 7, D-28359 Bremen, Germany; eleya@uni-bremen.de (N.E.); appiah@uni-bremen.de (C.A.); 2 MAPEX Center for Materials and Processes, University of Bremen, Bibliothekstraße 1, D-28359 Bremen, Germany; gesing@uni-bremen.de; 3 Institute of Inorganic Chemistry and Crystallography, University of Bremen, Leobener Straße 7, 28359 D-Bremen, Germany; enno.lork@uni-bremen.de (E.L.); mathias.gogolin@uni-bremen.de (M.G.); 4 Institute for Physical and Theoretical Chemistry, University of Bremen, Leobener Str. 7, D-28359 Bremen, Germany

**Keywords:** tetrels, group 14 elements, 11-membered rings, conformation

## Abstract

Unique eleven-membered rings containing silicon, germanium, and tin were synthesized in good yields by the reactions of the corresponding 1,2-bis((2-bromothiophen-3-yl)methoxy)benzenes with (C_6_H_5_)_2_ECl_2_ where E = Sn, Ge, Si. The Sn and Ge congeners were crystallized, but the conformers that these rings crystallized in, were quite different. As confirmed by Density Functional Theory (DFT) calculations, (C_28_H_22_O_2_S_2_Sn) assumes a unique crystal structure that leaves more room around the tetrel atom as compared to the crystal structure of the corresponding Ge compound. In the latter, the central cavity is quite open, whereas in the former, one of the methylene groups can fold inwards. Another consequence is the influence on the planes of the aromatic rings flanking the heterocycle. In the Ge case, the benzene ring is folded away from the central cavity, whereas in the Sn case, it is almost parallel to the imaginary axis through the center of the ring. Thermal analysis investigations (TGA and DSC methods) of these eleven-membered rings suggested the loss of a phenyl group in the first decomposition step. The decomposition temperature decreased from the Si containing heterocycle to Ge and was lowest for the Sn containing heterocycle.

## 1. Introduction

The chemistry of heterocyclic systems that contain heavier group 14 elements has received wide interest because of their special properties and reactivity [1,2,3]. For example, silicon, germanium, and tin heterocyclic compounds are widely used as the building units of polymers and oligomers [4,5,6,7,8]. The difference in size and bond lengths with these elements should make them useful for influencing the conformation of medium sized macrocycles, but such studies have not been performed yet.

**Scheme 1:**
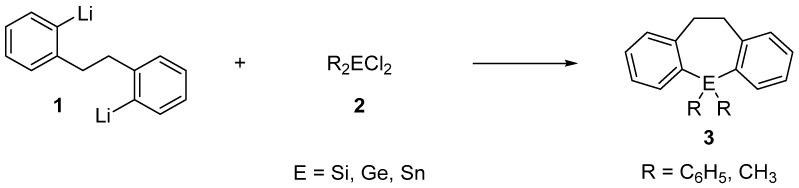
The synthetic route for dihydrodibenzo[b,f]metallepin derivatives.

A common method for preparing heterocyclic compounds containing main group elements is based on the nucleophilic substitution of halogen derivatives of the type R_2_ECl_2_ in which E is silicon, germanium, tin, or lead and R is a halogen or a hydrocarbon chain, with organo-magnesium or organo-lithium reagents [1,2,9,10]. For example, Corey et al. synthesized the first heavy element group 14 bridged heterocycle in 1972 by the reaction 2,2′-dilithiobibenzyl **1** with an appropriate Group IV halide **2** (Scheme 1) [11].

Although heterocyclic compounds containing group 14 elements are very advanced and can be designed using several synthetic approaches [12,13] they fall short of a detailed materials characterization. An important aspect to consider is the influence of the group 14 elements on the structural properties of the investigated molecule. The tetrel atoms differ substantially in size (C = 77 pm, Si = 111 pm, Ge = 125 pm, and Sn = 145 pm) as well as in bond length with carbon atoms (C–C = 154 pm, C–Si = 185 pm, C–Ge = 195 pm, and C–Sn = 216 pm) [14]. In 11-membered rings, which typically show ring strain, these two factors are likely to be pivotal for ring conformation and ring strain [15,16,17,18]. The tetrel series offers a unique probe for assessing these factors as there can be expected to be relatively little electronic contribution. Some of these questions have been addressed by us for other group 14 element compounds [19], regarding their thermal properties and conformational influences on the final structure. To date, there are no in-depth investigations on such medium sized ring systems containing a group 14 element and the possibilities this may offer for tuning the geometry.

Thus, we report three unique eleven-membered rings containing silicon, germanium, and tin by the reactions of the corresponding 1,2-bis((2-bromothiophen-3-yl)methoxy)benzenes with (C_6_H_5_)_2_ECl_2_ where (E = Sn, Ge, Si). The yields for these reactions were good throughout. In addition to NMR data, IR spectroscopy, and mass spectrometry, the three-dimensional molecular structures in the solid state could be determined with X-ray diffraction data for two of the three compounds. We also describe in detail how the tetrel substitution affects the thermal properties of the ring, with regards to their ring stability and their melting behavior, and confirm the results using Density Functional Theory (DFT) calculations [20,21].

## 2. Results and Discussion

### 2.1. Molecular Design and Synthesis

The basis for the study was the synthesis of 11-membered rings that differed only in the group 14 elements. The rings were designed to contain both rigid and flexible elements: rigid elements to reduce the number of possible conformations in order to arrive at interpretable data and overarching conclusions. Flexible elements were needed so that the rings could adopt different conformations to begin with. Because the only chemical difference of our target compounds was the use of the hetero elements Si, Ge, and Sn, it was important to introduce this feature at a very late stage of the synthesis. In this way, only one route had to be devised. Since these hetero elements should be most easily introduced in the form of electrophilic reagents of the type Ph_2_ECl_2_ (with E = Si, Ge, Sn), the other side was selected to be the aryl bromide, which can be easily transferred into a nucleophilic species by halogen–lithium exchange (Scheme 1).

**Scheme 2:**
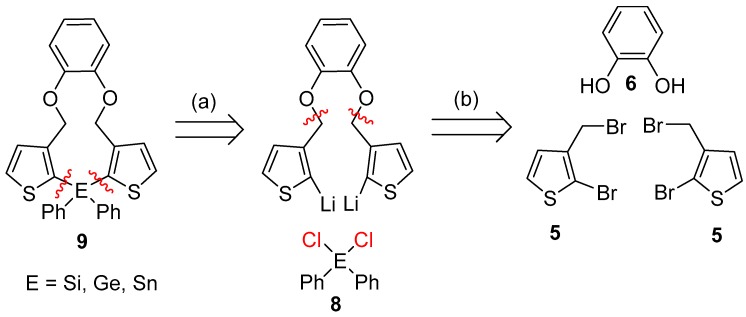
Retrosynthetic analysis for the synthesis of the target molecules.

Therefore, the retrosynthetic analysis required the dibrominated species **5,** which should be easily obtained by a Williamson etherification (Scheme 2).

**Scheme 3:**
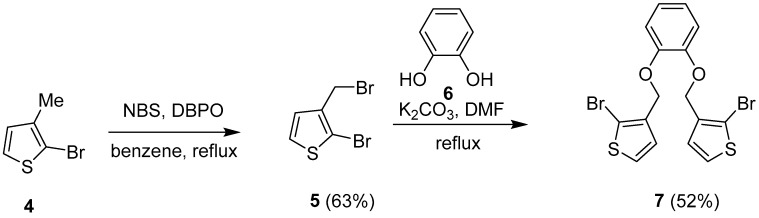
Synthesic route for 1,2-bis((2-bromothiophen-3-yl)methoxy)benzene **7**.

In the forward sense, a bromination reaction of the commercially available 2-bromo-3-methylthiophene **4** with *N*-bromosuccinimide (NBS) and dibenzoyl peroxide (DBPO), afforded 2-bromo-3-(bromomethyl)thiophene **5** in a yield of 63% (Scheme 3) [22]. Subsequently, the reaction of **5** using a protocol from Toppare and co-workers with 0.5 equivalents of catechol **6** afforded 1,2-bis((2-bromothiophen-3-yl)methoxy)benzene **7** in a yield of 52% [23].

**Scheme 4:**
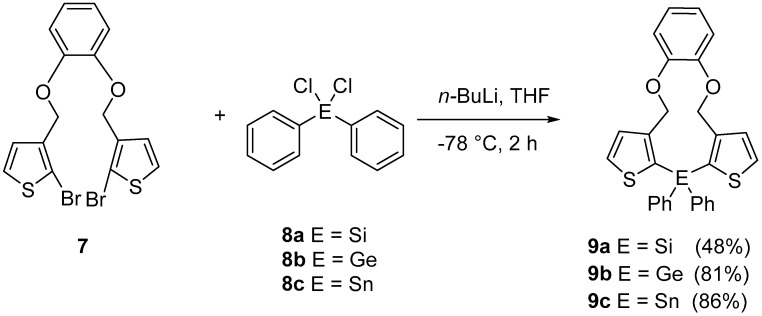
Synthesis of the eleven-membered rings containing the heavier tetrels Si, Ge, and Sn.

Subsequently, a facile halogen–lithium exchange of **7** using *n*-BuLi followed by treatment of the resulting organometallic intermediate with 1.0 equivalents of Ph_2_SiCl_2_, or Ph_2_GeCl_2_, or Ph_2_SnCl_2_, respectively, gave the products **9a**–**c** in good yields of 48% to 86%. (Scheme 4) [10].

### 2.2. Structural Analysis

**NMR Spectroscopy.** The chemical shifts of the tetrel atoms were: ^29^Si: −27.68 ppm and ^119^Sn: −161.94 ppm. By ^1^H NMR spectroscopy, the products **9a**–**c** are fairly similar concerning the chemical shifts. In all cases, the methylene group appeared as a singlet (**9a**: 5.00 ppm, **9b**: 5.02 ppm, **9c**: 5.13 ppm, all in DNSO-*d*_6_), which suggests a relatively free rotability of this group. This is in contrast to small rings, in which typically equatorial and axial substituents can be distinguished. Another remarkable feature is the chemical shift of the carbon atom in the thiophene rings directly bonded to the group 14 heteroatom. In **9a**, the shift is 133.95 ppm, whereas both for Ge and Sn, the shift is 145.98 ppm and 147.00 ppm, respectively. To rationalize this effect, the geometries of **9a**–**c** were optimized with Density Functional Theory (DFT) at the PBE/cc-pVDZ level of theory [24,25] to simulate the experimental geometries more closely. Starting from the crystal structure of **9b**, in **9a** the optimized bond length between the tetrel atom and the carbon atom in the thiophene ring decreases to 1.90 Å, which is shorter than the corresponding bond length in **9b** (1.97 Å) and **9c** (2.17 Å). Since the Si atom and the carbon atom of the thiophene ring are particularly close and Si has a slightly lower electronegativity than Ge and Sn, the carbon atom of the thiophene ring draws more electron density towards itself, resulting a lower chemical shift in **9a** as compared to **9b** and **9c**. A similar effect of bond length contraction accompanied by a decrease in chemical shift of an aromatic carbon atom connected to Si (1.89 Å, 142.95 ppm) as compared to Ge (2.02 Å, 146.12 ppm) and Sn (2.14 Å, 146.53 ppm) has been observed before [19], albeit slightly less significantly than in the present case.

**Figure 1:**
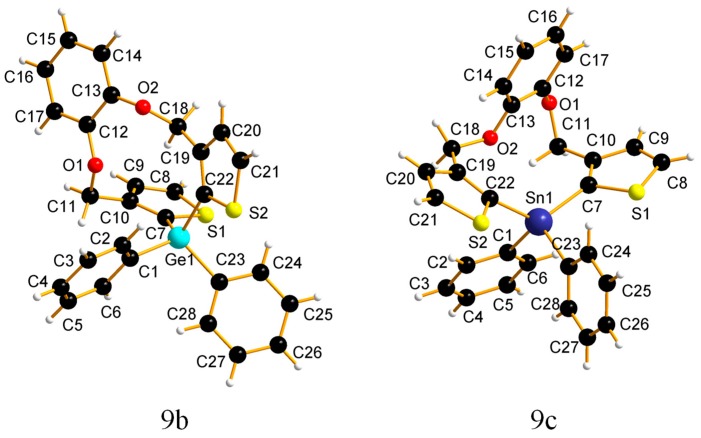
Molecular structures of compounds **9b** and **9c**, showing a 50% probability of ellipsoids.

**Figure 2:**
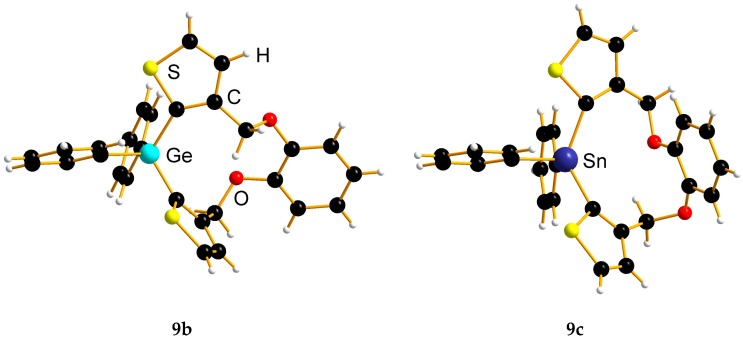
Molecular structures of compounds **9b** and **9c**. In **9b**, the CH_2_ group on C-18 is twisted inwards of the concave side of the molecule, whereas in **9c**, the overall larger ring size forces it to twist outwards, and thus opening up the cavity.

**Crystal structures.** The molecular structures of **9b** and **9c** are shown in Figure 1 and Figure 2 (for more images from different angles see the supporting info) to confirm the identity of the compounds and to showcase the major influence the heteroatom has on the conformation of the ring. The crystal structure of **9b** is triclinic and the density of the crystal is 1.51(1) g cm^−3^. On the other hand, the crystal structure of compound **9c** is monoclinic with a density of 1.606(2) g cm^−3^. Compound **9a** could not be crystalized. Selected bond lengths, angles, and torsion angles are collected in Table 1 and shown below.

From the crystal structure for compounds **9b** and **9c**, it can be observed that the distance of the carbon–tetrel bond increases from the lighter to the heavier element as expected. For compound **9b** the distances of the carbon–tetrel bond are (194.0(1) pm for (E1–C1), 194.4(1) pm for (E1–C7), 194.7(1) pm for (E1–C22), and 194.6(1) pm for (E1–C23) (for E = Ge)). The distances of the carbon–tetrel bond for compound **9c** are (213.1(2) pm for (E1–C1), 213.4(2) pm for (E1-C7), 213.2(2) pm for (E1–C22), and 214.6(2) pm for (E1–C23) (for E = Sn)). The differences in the bond angles between the carbon–tetrel–carbon atoms are (C22–E1–C7) 110.65(5)° (for E = Ge) and 123.99(7)° (for E = Sn). In addition, the torsion angles for the (C18–O2–C13–C12) are quite different 68.5(1)° (for **9b**) and –163.8(2)° (for **9c**), and the torsion angles for (C18–O2–C13–C14) are –116.7(1)° (for **9b**) and 14.2(3)° (for **9c**).

A major difference between the two structures is the conformation of the central cavity (Figure 2). In **9b**, the methylene group on C11 is twisted outwards and the methylene group based on C18 is twisted inwards. In the tin congener **9c**, the H atoms on both of these carbon atoms are pointing more (C18) or at least to some extent (C11) outwards. 

**DFT Calculations.** The influence of the heteroatom on the stability of the solid state structures and, in particular, on the conformation of the ring, was investigated with Density Functional Theory (DFT) [20,21] at the PBE/cc-pVDZ level of theory (calculation of the molecule in vacuum) [24,25]. The group 14 elements from carbon through tin were inserted into the experimental crystal structures of the compounds **9b** and **9c**, and the energy difference ΔE between the geometries at **9b** and **9c** was calculated. Hence, if ΔE is negative (or positive), the structure in the conformation of compound **9b** (or **9c**) would be more stable. The corresponding ΔE values are given in Table 2.

The ΔE values in Table 2 show that smaller group 14 elements favor the conformation of the **9b** crystal structure, whereas the **9c** structure becomes increasingly favorable with increasing atom size. This is because the C–E bonds become longer, which is the most decisive factor that determines which conformer is the most favorable. Tin is the only atom in the series that we investigated that favors the **9c** crystal structure, which matches the experimental findings. Since the experimental crystal structures were not re-optimized with DFT, errors in the bond lengths between the tetrel and the rest of the crystal structure arise, most severely in the case of carbon. However, these errors can be expected to largely cancel when calculating the ΔE values, since the same offset between the bond lengths in the crystal structure and the optimal value is included in **9b** and **9c**. Hence, the relative stabilities of the crystal structures and the conclusion that a larger atom size favors the crystal structure found in **9c** are supported by DFT.

In addition, the influence of the O2 atom on the tetrel atom was investigated by replacing this oxygen atom by a methylene group. Subsequently, the energy difference between the resulting **9b’** and **9c′** structures was calculated in the same way as in the native compounds. As can be seen in Table 2, the presence of the oxygen atom stabilizes the **9c** crystal structure, since the ΔE value generally decreases upon replacement of the oxygen atom by a methylene group. However, **9c’** still remains the favored structure in the case of tin, demonstrating that the proximity of oxygen atom 2 to the tin atom is a noticeable but not decisive factor for the stability of the **9c** crystal structure compared to **9b** if the central tetrel atom is tin.

Because intramolecular interactions may also influence how a material crystallizes, we performed a Hirshfeld analysis (see Supplementary Materials) [26,27,28,29]. From this, the C–H and H–H interactions dominate in both structures **9b** and **9c** and other effects are quite small.

### 2.3. Thermal Analysis of the Eleven-Membered Ring Systems

**Figure 3:**
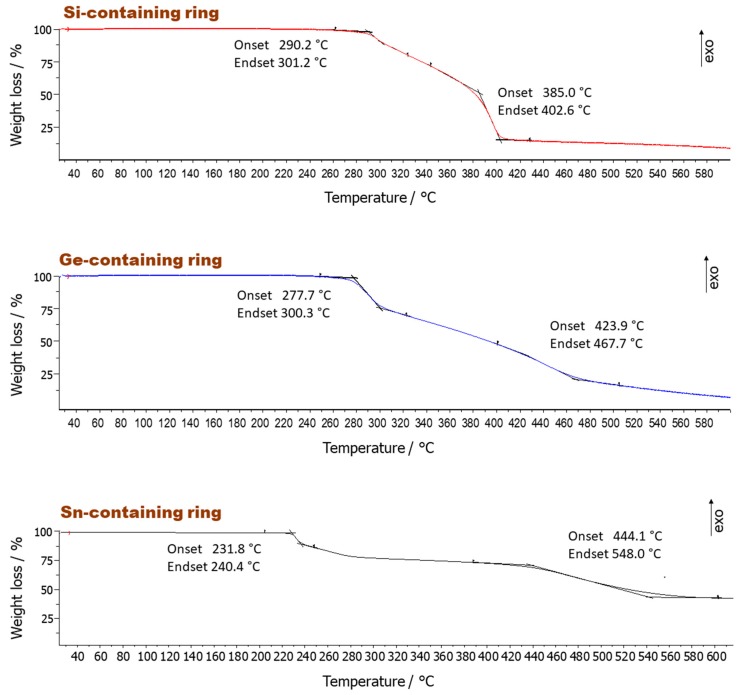
Thermogravimetric analysis (TGA) of the group 14 element ring systems (Si-containing ring **9a**, Ge-containing ring **9b**, and Sn-containing ring **9c**) at a heating rate of 10 K/min under nitrogen flow of 20 mL/min.

**Thermogravimetric Analysis (TGA):** The thermal decomposition of the synthesized compounds was investigated using thermogravimetric analysis (TGA). In general, two decomposition steps were observed for all investigated 11-membered rings in the range between 230 °C and 549 °C (Figure 3). The first of these was always steeper. 

The average values for the first decomposition step in the TGA were losses of 14% (**9a**), 12% (**9b**), and 13% (**9c**), respectively (Table 3). They are all very close to the ideal molar mass of the phenyl ring (calculated loss of C_6_H_5_ for **9a**: 16%, **9b**: 15%, **9c**: 13%), which suggests the release of a phenyl group attached to the group 14 elements. The onset temperature for the thermal decomposition (T_d1(onset)_) (Figure 3) also increased in the order of Sn (231 °C), Ge (278 °C), and Si (290 °C) ring systems. This is in accordance with the bond stability shown in Table 2 for these ring systems and previous investigations from Hoffmann et al. [19]. The Sn–C bond is more labile and thus prone to quicker thermal decomposition compared to the Si–C bond, hence proving the onset decomposition trend in Figure 3. It needs to be pointed out that the fragment loss can only result from the benzyl groups attached to the group 14 elements, based on the fact that the decomposition temperatures differ with different group 14 elements in the ring system. The other C_6_H_4_ group connected by the oxygen atoms was the same for all the ring systems and thus cleavage of this segment should result in nearly the same onset decomposition. Hence, it is likely that the 11-membered rings were comparatively stable after the first decomposition stage. 

Upon increasing the temperature in the TGA measurements, solid residues of the ring systems are expected to further decompose in the next decomposition step. However, this step was not sharp enough to elucidate the segments being removed. 

**Figure 4:**
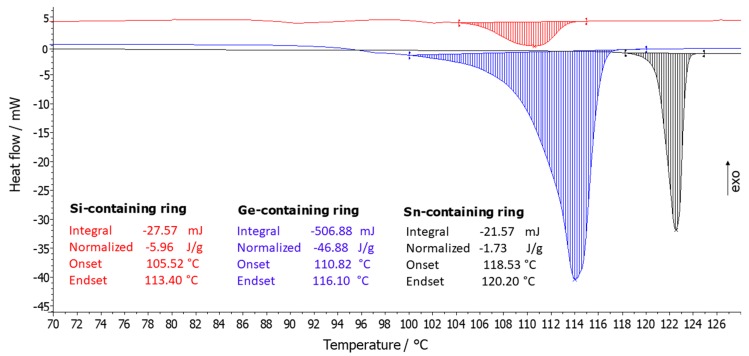
DSC heating scans at 10 K/min of the group 14 element ring systems (Sn-containing ring (**9c**), Ge-containing ring (**9b**), and Si-containing ring (**9a**)).

**Differential Scanning Calorimetry Analysis (DSC):** The thermal behavior of the organic ring molecules was evaluated using DSC. The heat flow curves with a linear temperature ramp of 10 °C/min for the Si (**9a**), Ge (**9b**), and Sn (**9c**) containing ring molecules (Figure 4) show melting peaks with onsets of 105.4 °C, 110.8 °C, and 118.5 °C (±0.2 °C). No crystallization peaks for these compounds were observed upon cooling at 10 K/min. When comparing the normalized melting enthalpies of the three different rings, we observed a clear difference between the three compounds. The Si and Sn-containing ring system showed a very small normalized melting enthalpy (normalized enthalpies: Si-ring = 5.96 J/g = 2.87 kJ/mol and Sn-ring = 1.73 J/g = 0.99 kJ / mol), with the Si-ring being slightly higher than the Sn-ring compound. However, the Ge-ring compound (normalized enthalpy = 46.88 J/g = 24.71 kJ/mol), had a much larger normalized melting enthalpy. The melting point temperatures (Figure 4) increased also in the order of Si-ring < Ge-ring < Sn-ring: 105.4 °C for the Si-ring to 110.8 °C for the Ge-ring, to 118.5 °C for the Sn-ring.

## 3. Materials and Methods

### 3.1. General Information

All reagents were purchased from commercial suppliers (Sigma-Aldrich, Merck KGaA, Darmstadt, Germany, Acros Organics, part of Thermo Fisher Scientific, The Hague, Netherlands, Apollo Scientific, Stockport, UK) and used without further purification. All reactions were carried out using standard Schlenk techniques under a dry, inert nitrogen atmosphere. All dry solvents were taken from a solvent purification system (SPS) from Inert Technology (Amesbury, MA, USA), degassed by three freeze-pump-thaw cycles, and stored under a nitrogen atmosphere unless noted otherwise.

^1^H NMR, ^13^C{^1^H} NMR, ^29^Si{^1^H} NMR, and ^119^Sn{^1^H} NMR spectra were recorded on a Bruker DRX 500 (Billerica, Massachusetts, USA) at 300 K. All ^1^H NMR and ^13^C{^1^H} NMR were referenced against the solvent residual proton signals (^1^H), or the solvent itself (^13^C). The ^29^Si{^1^H} NMR spectrum was referenced externally against tetramethylsilane. ^119^Sn{^1^H} NMR spectra were calculated based on the ^1^H NMR spectrum of tetramethylsilane. All chemical *δ* shifts are given in parts per million (ppm) and all coupling constants *J* in Hz. The exact assignment of the peaks was proved by two-dimensional NMR spectroscopy such as ^1^H COSY, ^13^C{^1^H} HSQC, or ^1^H/^13^C{^1^H} HMBC when possible.

Electron Impact (EI) ionization mass spectra were obtained on the double focusing mass spectrometer MAT 95+ or MAT 8200 from Finnigan Mat (Thermo Fisher, Waltham, MA, USA). Samples were measured by direct inlet or indirect inlet method with a source temperature of 200 °C. The ionization energy of the electron impact ionization was 70 eV. All signals were reported with the quotient from mass to charge *m*/*z*. High-resolution (HR) mass spectra were recorded on the double focusing mass spectrometer MAT 95+ from Finnigan Mat. Precision S7 weights were determined via the peak-matching method. The reference substance was perfluorokerosene (PFK). The resolution (R) of the peak-matching performance was 10,000. The calculated isotopic distribution for each ion agreed with experimental values.

IR spectra were recorded on a Nicolet Thermo iS10 Scientific IR spectrometer (Thermo Fisher, Waltham, MA, USA) with a diamond-ATR-unit. The resolution was 4 cm^−1^. Relative intensities of the IR bands were described by s = strong, m = medium, or w = weak.

All melting points were measured with a melting point apparatus by the company Gallenkamp (London, UK; the company is no longer operative) and are uncorrected.

Thermal analyses were performed on a standalone Mettler Toledo DSC 3+ STAR or a Mettler Toledo TGA/DSC 3+ system (Columbus, OH, USA), for which 40 µL and 100 µL aluminum crucibles were used. For TGA experiments, no lids were used, whereas for DSC experiments pierced lids were used.

Thin layer chromatography (TLC) was carried out on aluminum plates coated with silica gel 60 F254 with a layer thickness of 0.2 mm from Fluka (Honeywell, Bucharest, Romania) or Macherey-Nagel (Düren, Germany). All bands were detected by using a fluorescent lamp (254 nm and 366 nm). Column chromatography was carried out by using the column machine PuriFlash 4250 from Interchim (Montluçon, France). Silica gel columns of the type PF (PuriFlash) −15 (µm grain size) SiHP (Silica gel High Performance)-F0012 (gram), PF-15SiHP-F0025, PF-50SiHP-JP-F0080, and PF-50SiHP-JPF0120 were used. The sample was applied using a dry load method. The column material of the dry load was Celite 503 from Macherey-Nagel.

X-ray measurements were carried out at 100 K for compound **9b** and 83 K for compound **9c** on a Bruker Venture D8 diffractometer (Bruker, Karlsruhe, Germany) with Mo-Kα (71.07 pm) radiation. All structures were solved by intrinsic phasing and refined based on F^2^ by use of the SHELX program package, as implemented in OLex 1.2 [30]. All non-hydrogen atoms were refined using anisotropic displacement parameters. Hydrogen atoms attached to carbon atoms were included in geometrically calculated positions using a riding model. All crystals were obtained by slow evaporation of a heptane/ethanol mixture at 25 °C.

### 3.2. Computational Details

All calculations were conducted with the Q-Chem 5.2.1 program package [31]. The PBE/cc-pVDZ level of theory [24,25] was used in all calculations, except for the case of tin in which the cc-pVDZ-PP basis set, which includes an effective core potential and was taken from the Basis Set Exchange platform [32] was applied. Pure d-functions were applied throughout. The DIIS_GDM algorithm [33,34] was used to accelerate SCF convergence. The Avogadro program package was used to replace the oxygen atom 2 by a methylene group [35]. No further geometry optimization was carried out.

### 3.3. Syntheses

Synthesis of 2-Bromo-3-(bromomethyl)thiophene (**5**) [22]:
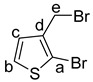


A suspension of 2-bromo-3-methylthiophene (**1**) (0.50 g, 2.8 mmol), NBS (0.50 g, 2.8 mmol), and benzoyl peroxide (7.0 mg, 0.28 mmol) in benzene (10 mL) was heated to reflux for 8 h. After allowing the mixture to cool to ambient temperature, the reaction mixture was filtered, and the precipitate was washed with pentane (30 mL). The organic solvent was removed under reduced pressure and the reaction mixture was purified by flash column chromatography using pentanes as eluent (R_f_ = 0.36) to give the desired product as a pale colorless oil (0.450 g, 63%). ^1^H NMR (500 MHz, CDCl_3_): 1H NMR (500 MHz, CDCl_3_): δ 7.25 (d, *J* = 5.7 Hz, 1H, H-b), 7.00 (d, *J* = 5.7 Hz, 1H, H-c), 4.45 (s, 2H, H-e) ppm. ^13^C{^1^H} NMR (126 MHz, CDCl_3_): δ 137.0 (C-a), 128.3 (CH-b), 126.4 (CH-b ), 113.3 (C-d), 25.7 (CH_2_-e) ppm. The NMR data are in agreement with the data found in the literature [22]. IR (ATR): *ṽ* = 3104 (w), 3026 (w), 2971 (w), 1749 (w), 1599 (w), 1514 (m), 1413 (s), 1347 (s), 1219 (s), 1186 (s), 1168 (s), 1101 (m), 971 (m), 900 (s), 849 (s), 773 (w), 722 (s), 636 (w), 555 (s) cm^−1^. HRMS (EI) *m*/*z* for C_5_H_4_^79^Br_2_S [M]^+^: calcd 253.8396, found: 253.83950.

Synthesis of 1,2-Bis((2-bromothiophen-3-yl)methoxy)benzene (**7**):
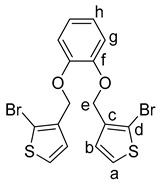


1,2-Dihydroxybenzene (**6**) (220 mg, 2.00 mmol) and anhydrous potassium carbonate (2.212 g, 16.00 mmol) were added into DMF (20 mL) in a two-necked flask under a nitrogen atmosphere. The mixture was stirred for one hour. 3-Bromomethylthiophene (1.0 g, 4.0 mmol) was added dropwise over the course of 10 min. The reaction mixture was heated to reflux for 8 h, and after cooling to 20 °C, the mixture was filtered and extracted with dichloromethane (3 × 30 mL). The combined organic layers were dried over magnesium sulfate, filtered, concentrated in vacuo, and purified by column chromatography (heptane:ethyl acetate 10:1; R_f_ = 0.25) to give the product **4** as white solid (0.96 mg, 52%); mp. 69–71 °C. ^1^H NMR (500 MHz, CDCl_3_): δ 7.61 (d, *J* = 5.6 Hz, 2H, H-a), 7.10 (d, *J* = 5.6 Hz, 2H, H-b), 7.08–7.03 (m, 2H, H-h), 6.96–6.89 (m, 2H, H-g), 4.99 (s,4H, H-e) ppm. ^13^C{^1^H} NMR (126 MHz, CDCl_3_): δ 148.07 (C-f), 137.11 (C-d), 128.62 (CH-b), 127.38 (CH-a), 121.70 (CH-g), 115.09 (CH-h), 111.21 (C-c), 64.69 (CH_2_-e) ppm. IR (ART): $\tilde{\nu }$ = 3112 (w), 2942 (w), 2872 (w), 1591 (m), 1498 (s), 1463 (s), 1455 (m), 1417 (s), 1379 (s), 1366 (m), 1326 (m), 1290 (s), 1246 (m), 1326 (w), 1246 (m), 1207 (s), 1123 (s), 1050 (s), 903 (s), 990 (s), 903 (s), 890 (m), 836 (s), 734 (s), 717 (s), 693 (m), 684 (m) cm^−1^. HR-MS (EI, C_16_H_12_O_2_S_2_^79^Br_2_; R = 10,000). Calcd: 457.86400. Found: 457.86347. MS (EI, 70 eV, direct inlet, 200 °C): *m*/*z* (% relative intensity) = 458 (5 ([M]**^+^**), 175 (100).

General Procedure for the Synthesis of Products (**9a**–**c**):

1,2-bis((2-bromothiophen-3-yl)methoxy)benzene **7** (100 mg, 217 µmol) was added to dry THF (10 mL) and cooled to −78 °C. To this a solution of 2.5 M *n*-BuLi in hexane (0.17 mL, 0.434 µmol) was added dropwise at −78 °C over a period of 5–10 min. Subsequently, the mixture was stirred at this temperature for 30 min. Then, Ph_2_SiCl_2_, or Ph_2_GeCl_2_, or Ph_2_SnCl_2_, (217 µmol, respectively) in THF (3.0 mL) was slowly added dropwise to the mixture. The mixture was further stirred at −78 °C for 2 h and then quenched at −78 °C with aq. H_2_O (30 mL) and extracted with CH_2_Cl_2_ (3 × 50 mL), washed with brine (3 × 50 mL), and dried over Na_2_SO_4_. After filtration, the solvent was removed in vacuo and the reaction mixtures were purified by column chromatography (heptane:ethyl acetate 9:1).

*15,15-Diphenyl-11,15-dihydro-4H-benzo[b]dithieno[3,2-f:2′,3′-i][1,4]dioxa[8]silacycloundecine* (**9a**):
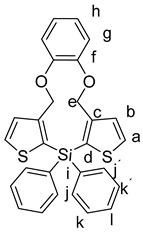


Starting with **7** (100 mg, 217 µmol), *n*-BuLi (2.5 M in hexane, 0.17 mL, 434 µmol), diphenyldichlorosilane **8a** (55 mg, 217 µmol), and THF (10 mL), **6a** was isolated as a colorless solid (49 mg, 46%); R_f_ = 0.27, mp 79–81 °C. ^1^H NMR (500 MHz, DMSO-*d_6_*): δ 7.87 (d, *J* = 4.7 Hz, 2H, H-a), 7.49 (m, 6H, H-k,k^´^,l), 7.46–7.39 (m, 4H,H-j,j^´^), 7.32 (d, *J* = 4.7 Hz, 2H, H-b), 7.12–6.96 (m, 2H, H-h), 6.92–6.78 (m, 2H, H-g), 5.00 (s, 4H, H-e) ppm. ^13^C{^1^H} NMR (126 MHz, DMSO-*d_6_*): δ 147.97 (C-f), 147.92 (C-c), 135.33 (CH-l), 133.95 (C-d), 133.23 (CH-a), 131.42 (CH-b), 130.18 (C-i), 130.16 (CH-k,k^´^,l), 127.98 (CH-j,j´), 122.57 (CH-h), 119.59 (CH-g), 67.27 (CH_2_-e) ppm. ^29^Si{^1^H}NMR (99 MHz, DMSO-*d_6_*): δ –27.68 ppm. IR (ART): *ṽ* = 3067 (w), 2925 (w), 1732 (w), 1588 (w), 1492 (s), 1463 (s), 1427 (s), 1405 (m), 1372 (m), 1355 (w), 1236 (s), 1185 (m), 1103 (s), 1040 (m), 1021 (m), 980 (m), 918 (w), 834 (w), 739 (m), 696 (s), 666 (w) cm^−1^. HR-MS (EI, C_28_H_22_SiO_2_S_2_; R = 10,000). Calcd: 482.08250 Found 482.08268. MS (EI, 70 eV, direct inlet, 200 °C): *m*/*z* (% relative intensity) = 482 (17 ([M]^+^), 213 (100).

*15,15-Diphenyl-11,15-dihydro-4H-benzo[b]dithieno[3,2-f:2′,3′-i][1,4]dioxa[8]germacycloundecine* (**9b**):
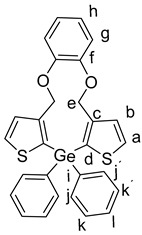


Starting with **7** (100 mg, 217 µmol), *n*-BuLi (2.5 M in hexane, 0.17 mL, 434 µmol), diphenylgermanium dichloride **8b** (65 mg, 0.217 µmol ), and THF (10 mL), **6b** was isolated as a colorless solid (93 mg, 81%); R_f_ *=* 0.23, mp. 114–115 °C. ^1^H NMR (500 MHz, DMSO-*d_6_*): δ 7.83 (d, *J* = 4.8 Hz, 2H, H-a), 7.51–7.40 (m, 10 H, H-j,j´,k,k´,l), 7.32 (d, *J* = 4.8 Hz, 2H, H-b), 7.00–6.95 (m, 2H, H-h), 6.85–6.81 (m, 2H, H-g), 5.02 (s, 4H, H-e) ppm. ^13^C{^1^H} NMR (126 MHz, DMSO-*d_6_*): δ 148.01(C-f), 145.98 (C-d), 135.84 (C-c), 134.23 (CH-a), 131.89 (CH-b), 131.19 (C-i), 130.87 (CH-j.j´), 129.68 (CH-k,k´), 128.41 (CH-l), 122.53 (CH-h), 119.24 (CH-g), 67.02 (CH_2_-e) ppm. IR (ART): *ṽ* = 3065 (w), 2864 (w), 1593 (w), 1522 (s), 1488 (m), 1465 (m), 1430 (s), 1409 (s), 1377 (m), 1355 (w), 1307 (w), 1273 (s), 1262 (s), 1250 (m), 1233 (s), 1250 (m), 1202 (m), 1182 (m), 1156 (m), 1106 (s), 1090 (s), 1068 (s), 1010 (s), 1041 (m), 977 (s), 921 (m), 853 (m), 734 (s), 721 (s), 693 (s), 660 (m) cm^−1^. HR-MS (EI, C_28_H_22_^70^GeO_2_S_2_; R = 10,000). Calcd: 524.02982. Found: 524.02996. MS (EI, 70 eV, direct inlet, 200 °C): *m*/*z* (% relative intensity) = 528 (20 ([M]^+^), 192 (100).

*15,15-Diphenyl-11,15-dihydro-4H-benzo[b]dithieno[3,2-f:2′,3′-i][1,4]dioxa[8]stannacycloundecine* (**9c**):
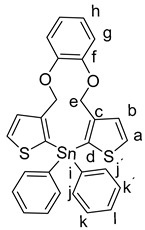


Starting with **7** (100 mg, 0.217 µmol), *n-*BuLi (2.5 M in hexane, 0.17 mL, 434 µmol), dichlorodiphenyltin **8c** (75 mg, 217 µmol), and THF (10 mL), **9c** was isolated as a colorless solid (107 mg, 86%); R_f_ *=* 0.22, mp. 132–134 °C. ^1^H NMR (500 MHz, DMSO-*d_6_*): δ 7.88 (d, *J* = 4.7 Hz, 2H, H-a), 7.62–7.51 (m, 4H, H -j,j´), 7.44–7.32 (m, 6H, H-k,k´,l), 7.31 (d, *J* = 4.7 Hz, 2H, H-b), 6.97–6.78 (m, 4H, H-g,h), 5.13 (s, 4H, H-e) ppm. ^13^C{^1^H} NMR (126 MHz, DMSO-*d_6_*): δ 147.94 (C-f), 147.00 (C-d), 138.82 (C-c), 136.26 (CH-a), 132.84 (CH-b), 130.66 (C-i), 129.30 (CH-j.j´), 129.08 (CH-k,k´), 128.41 (CH-l), 122.70 (CH-g), 118.45 (CH-h), 68.28 (CH_2_-e) ppm. ^119^Sn{^1^H} NMR (187 MHz, DMSO-*d_6_*): δ –161.94 ppm. IR (ART): *ṽ* = 3059 (w), 1588 (w), 1493 (s), 1458 (s), 1427 (w), 1401 (w), 1376 (w), 1306 (w), 1286 (w), 1286 (m), 1262 (w), 1235 (w), 1208 (m), 1193 (m), 1182 (w), 1110 (m), 1087 (m), 1073 (w), 1017 (w), 669 (m), 968 (w), 907 (w), 825 (w), 815 (w), 740 (s), 722 (s), 697 (s), 657 (m) cm^−1^. HRMS (ESI; C_28_H_22_O_2_S_2_^120^ Sn + H)^+^: Calcd: 575.01559. Found: 575.01606. ). MS (ESI): *m*/*z* = 612 (90 [M + K]^+^), 596 (100 [M + Na]^+^), 575 (20 [M + H]^+^).

## 4. Conclusions

In conclusion, we reported a facile and efficient approach to the synthesis of new and stable eleven-membered ring compounds containing the group 14 elements silicon, germanium, and tin in good yields. These compounds crystallize in different crystal structures with very different conformers. This can be directly attributed to the influence of the size and bond lengths to the tetrel atom, as demonstrated by Density Functional Theory (DFT) calculations. The central cavity for the lighter elements is very open, whereas a methylene group can fold over it in the Sn-case. Thermal investigations of these eleven-membered ring compounds using TGA and DSC revealed that the central ring system is stable in the first decomposition step. Accordingly, the normalized melting enthalpies of the three different ring systems were for the Si-ring = 5.96 J/g, the Sn-ring = 1.73 J/g and for the Ge-ring = 46.88 J/g, with increased onset melting temperatures from Si-ring (105.4 °C), to Ge-ring (110.8 °C), to Sn-ring (118.5 °C).

The increase of melting enthalpy shows that the lattice energy of the Ge containing ring is higher than those of the Si compound. Therefore, the higher melting temperature could be interpreted as a side effect related to the melting enthalpy, which is correlated to the lattice energy. Taking the increase of the melting enthalpy from the Si to the Ge containing compound into account, the corresponding enthalpy of the Sn containing phase could therefore be expected to be higher. However, it was observed to be lower because the Sn system evades this increase in energy by crystallizing in an entirely different crystal structure.

## Figures and Tables

**Table 1:** Selected interatomic distances and angles of **9b** and **9c,** and torsion angles.

	9b (E = Ge)	9c (E = Sn)
	Bond Lengths/pm	
E1–C1	194.0(1)	213.1(2)
E1–C7	194.4(1)	213.4(2)
E1–C22	194.7(1)	213.2(2)
E1–C23	194.6(1)	214.6(2)
	Bond Angels/°	
C1–E1–C7	112.56(5)	110.78(7)
C1–E1–C22	108.82(5)	104.33(7)
C1–E1–C23	109.72(5)	109.98(7)
C7–E1–C23	107.94(5)	103.67(7)
C22–E1–C7	110.65(5)	123.99(7)
C22–E1–C23	107.01(5)	103.47(7)
C13–O2–C18	114.20(1)	117.35(1)
O1–C12–C13	116.20(1)	122.18(1)
O1–C12–C17	124.10(1)	117.70(1)
O2–C13–C12	120.60(1)	115.74(1)
O2–C13–C14	119.20(1)	124.60(1)
O2–C18–C19	107.80(1)	113.16(1)
	Torsion Angels/°	
C7–E1–C1–C2	−131.7(1)	−156.3(1)
C7–E1–C1–C6	50.0(1)	27.3(2)
C22–E1–C1–C2	−8.7(1)	−20.7(2)
C22–E1–C1–C6	173.1(1)	162.9(2)
C1–E1–C7–S1	−158.5(7)	−128.3(1)
C22–E1–C7–S1	79.4(8)	106.6(1)
C1–E1–C22–S2	77.6(8)	107.1(1)
C7–E1–C22–S2	−158.2(7)	−125.0(1)
C23–E1–C22–S2	−40.8(8)	−8.0(1)
C23–E1–C22–C19	147.0(1)	−172.4(2)
C11–O1–C12–C13	−147.2(1)	−56.9(2)
C11–O1–C12–C17	39.1(2)	129.7(2)
C18–O2–C13–C12	68.5(1)	−163.8(2)
C18–O2–C13–C14	−116.7(1)	14.2(3)

**Table 2:** Energy differences ΔE (kJ/mol) (calculated by DFT) between the crystal structures of compounds **9b** and **9c** with different group 14 elements (native), as well as analogous structures **9b’** and **9c’** in which oxygen atom 2 was replaced by methylene groups.

Atom	C	Si	Ge	Sn
**ΔE (kJ/mol), native**	−312.9	−131.8	−65.5	172.8
**ΔE (kJ/mol), methylene**	−359.8	−190.5	−127.1	103.2

**Table 3:** Decomposition (T_d(onset)_, T_d(endset)_, and percent lost segments), Melting temperatures (T_m_) and normalized enthalpy of melting of the rings containing Si (**9a**), Ge (**9b**), and Sn (**9c**).

	TGA (Accuracy ± 0.5 °C)			DSC (Accuracy ± 0.2 °C)			
Sample	T_d1(onset)_/°C	T_d1f(endset)_/°C	Loss/%	T_m(onset)_/°C	T_m(endset)_/°C	Normalized Enthalpy	
						/(J/g)	/(kJ/mol)
**Si-Ring (6a)**	290.2	302.2	14	105.4	113.4	5.96	2.87
**Ge-Ring (6b)**	277.6	300.3	12	110.8	116	46.88	24.71
**Sn-Ring (6c)**	231.8	240.4	13	118.5	120.7	1.73	0.99

T_d1_ represents the first thermal decomposition step for each ring system, T_d1f_ represents the first endset of decomposition, T_m_ is the melting temperature.

## Supplementary Materials

The following are available online: A list of all reagents and solvents, attempted syntheses, images of all NMR spectra, images of the data of all thermoanalytical experiments (DSC and TGA).
