# Supplementary material for: Synthesis and Thermal Investigations of Eleven-Membered Ring Systems Containing One of the Heavier Group 14 Element Atoms Si, Ge, and Sn
Source: Molecules. 2020 Jan 10;25(2):283. doi: 10.3390/molecules25020283 (PMC7024359; doi:10.3390/molecules25020283)
Supplement: Supplementary file 1 [file molecules-25-00283-s001.pdf]

Supporting Information

for

Synthesis and Thermal Investigations of Eleven-Membered Ring Systems Containing one of the Heavier Group 14 Element Atoms Si, Ge, and Sn

Nadi Eleya [1,2], Clement Appiah [1,2], Enno Lork [3], Mathias Gogolin [3], Thorsten M. Gesing [2,3], Tim Stauch[2,4,*] and Anne Staubitz [1,2,*]

[1] University of Bremen, Institute for Analytical and Organic Chemistry, Leobener Straße 7, D-28359 Bremen, Germany; eleya@uni-bremen.com (N.E); appiah@uni-bremen.de (C.A); staubitz@uni-bremen.de (+A.S.)

[2] University of Bremen, MAPEX Center for Materials and Processes, Bibliothekstraße 1, D-28359 Bremen, Germany

[3] University of Bremen, Institute of Inorganic Chemistry and Crystallography, Leobener Straße 7, 28359 D-Bremen, Germany; enno.lork@uni-bremen.de (E.L.); mathias.gogolin@uni-bremen.de (M.G.); gesing@uni-bremen.de (T.M.G.)

[4] University of Bremen, Institute for Physical and Theoretical Chemistry, Leobener Str. 7, D-28359 Bremen, Germany; tstauch@uni-bremen.de (T.S.)

* Correspondence: staubitz@uni-bremen.com; Tel.: +49-431-218-63210

Table of Contents

1. General considerations 3

 1.1 Abbreviations 3

 1.2 Chemicals and solvents 4

2. Syntheses 7

3. NMR spectra of all reported structures 12

 Figure (1) ^{1}H NMR (500 MHz) spectrum of 5 in $CDCl_3$ 12

 Figure (2) $^{13}C\{^{1}H\}$ NMR (126 MHz) spectrum of 5 in $CDCl_3$ 12

 Figure (3) ^{1}H NMR (500 MHz) spectrum of 7 in DMSO-d_6 13

 Figure (4) $^{13}C\{^{1}H\}$ NMR (126 MHz) spectrum of 7 in DMSO-d_6 13

 Figure (5) ^{1}H NMR (500 MHz) spectrum of 9a in DMSO-d_6 14

 Figure (6) $^{13}C\{^{1}H\}$ NMR (126 MHz) spectrum of 9a in DMSO-d_6 14

 Figure (7) $^{29}Si\{^{1}H\}$ NMR (119 MHz) spectrum of 9a in DMSO-d_6 15

 Figure (8) ^{1}H NMR (500 MHz) spectrum of 9b in DMSO-d_6 15

 Figure (9) $^{13}C\{^{1}H\}$ NMR (126 MHz) spectrum of 9b in DMSO-d_6 16

 Figure (10) ^{1}H NMR (500 MHz) spectrum of 9c in DMSO-d_6 16

 Figure (11) $^{13}C\{^{1}H\}$ NMR (126 MHz) spectrum of 9c in DMSO-d_6 17

 Figure (12) $^{119}Sn\{^{1}H\}$ NMR (187 MHz) spectrum of 9c in DMSO-d_6 17

4. Further visualizations of structures 9b and 9c to illustrate the geometrical differences of the conformers 18

 Figure (13) Shape of 9b; H were omitted for clarity. 18

 Figure (14) Shape of 9c; H were omitted for clarity. 18

5. Hirshfeld surface analysis 19

 Figure (15) Fingerprint plots for 9b and 9c. External atom distances to the Hirshfeld surface de against internal atom distance d_i 19

 Figure (16) The relative coverage of the Hirshfeld surface 20

 Figure (17) The Hirschfeld surface of 9b (left) and 9c (right) colour-coded for electrostatic potential. Red for negative and blue for positive. 21

6. Summary of the crystal data for compounds 9b and 9c 21

7. Bibliography 23

1. General considerations

1.1 Abbreviations

ATR	Attenuated total reflection
calcd.	calculated
COSY	Correlated spectroscopy
d	Doublet (NMR)
dd	Doublet of doublets (NMR)
DFS	Double-focusing sector field
DMF	*N*,*N*-Dimethylformamide
DMSO	*N*,*N*-Dimethyl sulfoxide
EI	Electron ionization
ESI	Electrospray ionization
HPLC	High pressure liquid chromatography
HRMS	High resolution MS
HMBC	Heteronuclear Multiple Bond Correlation
HSQC	Heteronuclear Single Quantum Coherence
IR	Infrared spectroscopy
IT	Ion trap
J	Coupling constant (NMR)
LED	Light-emitting diode
m	Medium (IR)
m	multiplet (in NMR spectroscopy)
mp	Melting point

MS	Mass spectrometry
NMR	Nuclear magnetic resonance spectroscopy
ppm	Parts per million
R	Resolution
s	Singlet (NMR), strong (IR)
SPS	Solvent purification system
t	Triplet (NMR)
TLC	Thin layer chromatography
UV-Vis.	Ultra violet and visible light spectroscopy
w	Weak (IR)

1.2 Chemicals and solvents

All reactions were carried out using standard Schlenk techniques under a dry, inert nitrogen atmosphere or inside a nitrogen filled glovebox from Inert, Innovative Technology, Inc. Company (< 0.1 ppm O2 and < 0.1 ppm H_2O) unless noted otherwise. All dry solvents were taken from the solvent purification system (SPS), degassed by three freeze-pump-thaw cycles and stored under a nitrogen atmosphere unless noted otherwise. All chemicals were commercially available and were used without further purification unless noted otherwise.

Table 1: List of suppliers and purities of the chemicals used.

Reagent	**Supplier**	**Purity**	**Comments**
2-Bromo-3-methylthiophene	Apollo Scientific	97%	
Dibenzoyl peroxide	Tokio Chemical Industry	Wetted With ca. 25% water	2.5 M in hexanes
Diphenyltin dichloride	VWR Chemicals	96% distilled	stored in a freezer inside the glove box
Diphenylgermanium dichloride	Sigma Aldrich	98%	stored in a freezer inside the glove box
Diphenyldichlorosilane	Alfa Aesar	97%	stored in a freezer

			inside the glove box
Magnesium sulfate	Grüssing	99%	
n-Butyllithium	Acros Organics	n. a.	2.5 M in hexanes
N-bromosuccinimide	MERCK	99%	
Potassium carbonate	Sigma Aldrich	99%	

Table 2: List of suppliers and purity of the solvents used.

**Solvent**	**Comments**
Dichloromethane	VWR Chemicals, 99.9%
Dimethylformamide	Extra dry from Acros Organics, 99.8%
Ethyl acetate	Fischer Chemicals, 99.97%
n-Pentane	VWR Chemicals; Evaporation
Tetrahydrofuran	VWR Chemicals; dry from the SPS and degassed

1.3 Analytical instruments

^{1}H NMR, ^{13}C{^{1}H} NMR, ^{29}Si{^{1}H} NMR, and ^{119}Sn{^{1}H} NMR spectra were recorded on a Bruker DRX 500 at 300 K. All 1H NMR and ^{13}C{^{1}H} NMR were referenced against the solvent residual proton signals (^{1}H), or the solvent itself (^{13}C). The ^{29}Si{^{1}H} NMR spectrum was referenced externally against tetramethylsilane. The ^{119}Sn{^{1}H} NMR spectrum was calculated based on the ^{1}H NMR spectrum of tetramethylsilane. All chemical δ shifts are given in parts per million (ppm) and all coupling constants *J* in Hz. The exact assignment of the peaks was proved by two-dimensional NMR spectroscopy such as ^{1}H COSY, ^{13}C{^{1}H} HSQC or ^{1}H/^{13}C{^{1}H} HMBC when possible.

Electron Impact (EI) ionization mass spectra were obtained on the double focusing mass spectrometer MAT 95+ or MAT 8200 from Finnigan Mat. Samples were measured by direct inlet or indirect inlet method with a source temperature of 200 °C. The ionization energy of the electron impact ionization was 70 eV. All signals were reported with the quotient from mass to charge m/z. High-resolution (HR) mass spectra were recorded on the double focusing mass spectrometer MAT 95+ from Finnigan Mat. Precision S7 weights were determined via the peak-matching method. The reference substance was perfluorokerosene (PFK). The resolution (R) of the peak-matching performance was 10000. The calculated isotopic distribution for each ion agreed with experimental values.

IR spectra were recorded on a Nicolet Thermo iS10 Scientific IR spectrometer with a diamond-ATR-unit. The resolution was 4 cm^{-1}. Relative intensities of the IR bands were described by s = strong, m = medium or w = weak.

All melting points were measured with a melting point apparatus by the company Gallenkamp and are uncorrected.

Thermal analyses were performed on a standalone Mettler Toledo DSC 3+ STAR or a Mettler Toledo TGA/DSC 3+ system, for which 40 µL and 100 µL aluminum crucibles were used. For TGA experiments, no lids were used, whereas for DSC experiments, pierced lids were used.

Thin layer chromatography (TLC) was carried out on aluminum plates coated with silica gel 60 F254 with a layer thickness of 0.2 mm from Fluka or Macherey-Nagel. All bands were detected by using a fluorescent lamp (254 nm and 366 nm). Column chromatography was carried out by using the column machine PuriFlash 4250 from Interchim. Silica gel columns of the type PF (PuriFlash) -15 (µm grain size) SiHP (Silica gel High Performance) -F0012 (gram), PF-15SiHP-F0025, PF-50SiHP-JP-F0080, and PF-50SiHP-JPF0120. were used. The sample was applied using a dry load method. The column material of the dry load was Celite 503 from Macherey-Nagel.

X-ray measurements were carried out at 100 K for compound 9b and 83 K for compound 9c on a Bruker Venture D8 diffractometer (Bruker, Karlsruhe, Germany) with Mo-Kα (71.07 pm) radiation. All structures were solved by intrinsic phasing and refined based on F2 by use of the SHELX program package, as implemented in OLex 1.2.[1] All non-hydrogen atoms were refined using anisotropic displacement parameters. Hydrogen atoms attached to carbon atoms were included in geometrically calculated positions using a riding model. All crystals were obtained by slow evaporation of a heptane/ethanol mixture at 25 °C.

2. Syntheses

Synthesis of 2-bromo-3-(bromomethyl)thiophene (5).[2]

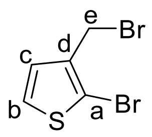

A suspension of 2-bromo-3-methylthiophene (**1**) (0.50 g, 2.8 mmol), NBS (0.50 g, 2.8 mmol) and benzoyl peroxide (7.0 mg, 0.28 mmol) in benzene (10 mL) was heated to reflux for 8 h. After allowing the mixture to cool to ambient temperature, the reaction mixture was filtered, and the precipitate was washed with pentane (30 mL). The organic solvent was removed under reduced pressure and the reaction mixture was purified by flash column chromatography using pentanes as eluent (R_f = 0.36) to give the desired product as a pale colorless oil (0.450 g, 63%). ^{1}H NMR (500 MHz, $CDCl_3$): ^{1}H NMR (500 MHz, $CDCl_3$): δ 7.25 (d, *J* = 5.7 Hz, 1H, H-b), 7.00 (d, *J* = 5.7 Hz, 1H, H-c), 4.45 (s, 2H, H-e) ppm. ^{13}C{^{1}H} NMR (126 MHz, $CDCl_3$): δ 137.0 (C-a), 128.3 (CH-b), 126.4 (CH-b), 113.3 (C-d), 25.7 (CH_2-e) ppm. The NMR data are in agreement with the data found in the literature.[2] IR (ATR): $\tilde{\nu}$ = 3104 (w), 3026 (w), 2971 (w), 1749 (w), 1599 (w), 1514 (m), 1413 (s), 1347 (s), 1219 (s), 1186 (s), 1168 (s), 1101 (m), 971 (m), 900 (s), 849 (s), 773 (w), 722 (s), 636 (w), 555 (s) cm^{-1}. HRMS (EI) *m/z* for $C_5H_4{}^{79}Br_2S$ $[M]^+$: calcd 253.8396, found: 253.83950.

Synthesis of 1,2-bis((2-bromothiophen-3-yl)methoxy)benzene (7)

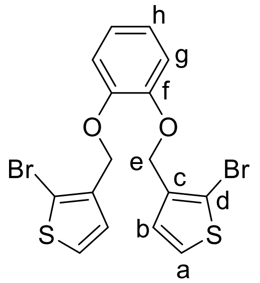

1,2-Dihydroxybenzene (**6**) (220 mg, 2.00 mmol) and anhydrous potassium carbonate (2.212 g, 16.00 mmol) were added into DMF (20 mL) in a two necked flask under a nitrogen atmosphere. The mixture was stirred for one hour. 3-Bromomethylthiophene (1.0 g, 4.0 mmol) was added dropwise over the course of 10 min. The reaction mixture was heated to reflux for 8 h, and after cooling to 20 °C, the mixture was filtered and extracted with dichloromethane (3 x 30 mL). The combined organic layers were dried over magnesium

sulfate, filtered, concentrated in vacuo and purified by column chromatography (heptane : ethyl acetate 10 : 1 R_f = 0.25) to give the product **4** as white solid (0.96 mg, 52 %); mp. 69-71 °C. ^{1}H NMR (500 MHz, $CDCl_3$): δ 7.61 (d, *J* = 5.6 Hz, 2H, H-a), 7.10 (d, *J* = 5.6 Hz, 2H, H-b), 7.08 – 7.03 (m, 2H, H-h), 6.96 – 6.89 (m, 2H, H-g), 4.99 (s,4H, H-e) ppm. ^{13}C{^{1}H} NMR (126 MHz, $CDCl_3$): δ 148.07 (C-f), 137.11 (C-d), 128.62 (CH-b), 127.38 (CH-a), 121.70 (CH-g), 115.09 (CH-h), 111.21 (C-c), 64.69 (CH_2-e) ppm. IR (ART): $\tilde{\nu}$ = 3112 (w), 2942 (w), 2872 (w), 1591 (m), 1498 (s), 1463 (s), 1455 (m), 1417 (s), 1379 (s), 1366 (m), 1326 (m), 1290 (s), 1246 (m), 1326 (w), 1246 (m), 1207 (s), 1123 (s), 1050 (s), 903 (s), 990 (s), 903 (s), 890 (m), 836 (s), 734 (s), 717 (s), 693 (m), 684 (m) cm^{-1}. HR-MS (EI, $C_{16}H_{12}O_2S_2{}^{79}Br_2$; R = 10000). Calcd: 457.86400. Found: 457.86347. MS (EI, 70 eV, direct inlet, 200 °C): *m/z* (% relative intensity) = 458 (5 ($[M]^+$), 175 (100).

General procedure for the synthesis of products (9a-c):

1,2-bis((2-bromothiophen-3-yl)methoxy)benzene **7** (100 mg, 217 µmol) was added to dry THF (10 mL) and cooled to -78 °C. To this a solution of 2.5 M *n*-BuL in hexane (0.17 mL, 0.434 µmol) was added dropwise at -78 °C over a period of 5-10 min. Subsequently, the mixture was stirred at this temperature for 30 min. Then, Ph_2SiCl_2, or Ph_2GeCl_2, or Ph_2SnCl_2, (217 µmol, respectively) in THF (3.0 mL) was slowly added dropwise to the mixture. The mixture was further stirred at -78 °C for 2 h and then quenched at -78 °C with aq. H_2O (30 mL) and extracted with CH_2Cl_2 (3 x 50 mL), washed with brine (3 x 50 mL) and dried over Na_2SO_4. After filtration, the solvent was removed *in vacuo* and the reaction mixtures were purified by column chromatography (heptane : ethyl acetate 9 : 1).

15,15-Diphenyl-11,15-dihydro-4H-benzo[b]dithieno[3,2-f:2',3'-i][1,4]dioxa[8]silacycloundecine (9a)

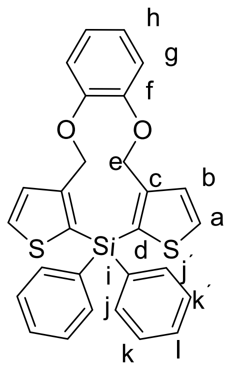

Starting with **7** (100 mg, 217 µmol), *n*-BuLi (2.5 M in hexane, 0.17 mL, 434 µmol), diphenyldichlorosilane **8a** (55 mg, 217 µmol), and THF (10 mL), **6a** was isolated as a colorless solid (49 mg, 46%); R_f = 0.27, mp 79-81 °C. ^{1}H NMR (500 MHz, DMSO-*d*$_6$): δ 7.87 (d, *J* = 4.7 Hz, 2H, H-a), 7.49 (m, 6H, H-k,k´,l), 7.46 – 7.39 (m, 4H,H-j,j´), 7.32 (d, *J* = 4.7 Hz, 2H, H-b), 7.12 – 6.96 (m, 2H, H-h), 6.92 – 6.78 (m, 2H, H-g), 5.00 (s, 4H, H-e) ppm. ^{13}C{^{1}H} NMR (126 MHz, DMSO-*d*$_6$): δ 147.97 (C-f), 147.92 (C-c), 135.33 (CH-l), 133.95 (C-d), 133.23 (CH-a), 131.42 (CH-b), 130.18 (C-i), 130.16 (CH-k,k´,l), 127.98 (CH-j,j´), 122.57 (CH-h), 119.59 (CH-g), 67.27 (CH_2-e) ppm. ^{29}Si{^{1}H}NMR (99 MHz, DMSO-*d*$_6$): δ -27.68 ppm. IR (ART): $\tilde{\nu}$ = 3067 (w), 2925 (w), 1732 (w), 1588 (w), 1492 (s), 1463 (s), 1427 (s), 1405 (m), 1372 (m), 1355 (w), 1236 (s), 1185 (m), 1103 (s), 1040 (m), 1021 (m), 980 (m), 918 (w), 834 (w), 739 (m), 696 (s), 666 (w) cm^{-1}. HR-MS (EI, $C_{28}H_{22}SiO_2S_2$; R = 10000). Calcd: 482.08250 Found 482.08268. MS (EI, 70 eV, direct inlet, 200 °C): *m/z* (% relative intensity) = 482 (17 ([M]$^+$), 213 (100).

15,15-Diphenyl-11,15-dihydro-4H-benzo[b]dithieno[3,2-f:2',3'-i][1,4]dioxa[8]germacycloundecine (9b)

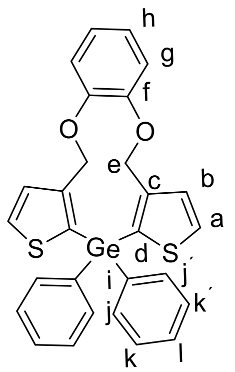

Starting with **7** (100 mg, 217 µmol), *n*-BuLi (2.5 M in hexane, 0.17 mL, 434 µmol), diphenylgermanium dichloride **8b** (65 mg, 0.217 µmol), and THF (10 mL), **6b** was isolated as a colorless solid (93 mg, 81 %); R_f = 0.23, mp. 114-115 °C. ^{1}H NMR (500 MHz, DMSO-d_6): δ 7.83 (d, *J* = 4.8 Hz, 2H, H-a), 7.51 – 7.40 (m, 10 H, H-j,j´,k,k´,l), 7.32 (d, *J* = 4.8 Hz, 2H, H-b), 7.00 – 6.95 (m, 2H, H-h), 6.85 – 6.81 (m, 2H, H-g), 5.02 (s, 4H, H-e) ppm. ^{13}C{^{1}H} NMR (126 MHz, DMSO-d_6): δ 148.01(C-f), 145.98 (C-d), 135.84 (C-c), 134.23 (CH-a), 131.89 (CH-b), 131.19 (C-i), 130.87 (CH-j.j´), 129.68 (CH-k,k´), 128.41 (CH-l), 122.53 (CH-h), 119.24 (CH-g), 67.02 (CH_2-e) ppm. IR (ART): $\tilde{\nu}$ = 3065 (w), 2864 (w), 1593 (w), 1522 (s), 1488 (m), 1465 (m), 1430 (s), 1409 (s), 1377 (m), 1355 (w), 1307 (w), 1273 (s), 1262 (s), 1250 (m), 1233 (s), 1250 (m), 1202 (m), 1182 (m), 1156 (m), 1106 (s), 1090 (s), 1068 (s), 1010 (s), 1041 (m), 977 (s), 921 (m), 853 (m), 734 (s), 721 (s), 693 (s), 660 (m) cm^{-1}. HR-MS (EI, $C_{28}H_{22}{}^{70}GeO_2S_2$; R = 10000). Calcd: 524.02982. Found: 524.02996. MS (EI, 70 eV, direct inlet, 200 °C): *m/z* (% relative intensity) = 528 (20 ($[M]^+$), 192 (100).

15,15-Diphenyl-11,15-dihydro-4H-benzo[b]dithieno[3,2-f:2',3'-i][1,4]dioxa[8]stannacycloundecine (9 c)

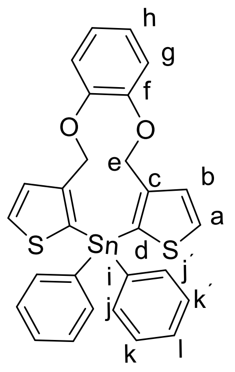

Starting with **7** (100 mg, 0.217 µmol), *n*-BuLi (2.5 M in hexane, 0.17 mL, 434 µmol), Dichlorodiphenyltin **8c** (75 mg, 217 µmol), and THF (10 mL), **9c** was isolated as a colorless solid (107 mg, 86 %); R_f = 0.22, mp. 132-134 °C. ^{1}H NMR (500 MHz, DMSO-d_6): δ 7.88 (d, *J* = 4.7 Hz, 2H, H-a), 7.62 – 7.51 (m, 4H, H -j,j´), 7.44 – 7.32 (m, 6H, H-k,k´,l), 7.31 (d, *J* = 4.7 Hz, 2H, H-b), 6.97 – 6.78 (m, 4H, H-g,h), 5.13 (s, 4H, H-e) ppm. ^{13}C{^{1}H} NMR (126 MHz, DMSO-d_6): δ 147.94 (C-f), 147.00 (C-d), 138.82 (C-c), 136.26 (CH-a), 132.84 (CH-b), 130.66 (C-i), 129.30 (CH-j.j´), 129.08 (CH-k,k´), 128.41 (CH-l), 122.70 (CH-g), 118.45 (CH-h), 68.28 (CH_2-e) ppm. ^{119}Sn{^{1}H} NMR (187 MHz, DMSO-d_6): δ -161.94 ppm. IR (ART): $\tilde{\nu}$ = 3059 (w), 1588 (w), 1493 (s), 1458 (s), 1427 (w), 1401 (w), 1376 (w), 1306 (w), 1286 (w), 1286 (m), 1262 (w), 1235 (w), 1208 (m), 1193 (m), 1182 (w), 1110 (m), 1087 (m), 1073 (w), 1017 (w), 669 (m), 968 (w), 907 (w) , 825 (w), 815 (w), 740 (s), 722 (s), 697 (s), 657 (m) cm^{-1}. HRMS (ESI; $C_{28}H_{22}O_2S_2{}^{120}Sn+H)^+$: Calcd: 575.01559. Found: 575.01606.). MS (ESI): *m/z* = 612 (90 $[M + K]^+$), 596 (100 $[M + Na]^+$), 575 (20 $[M + H]^+$).

3. NMR spectra of all reported structures

Figure (1) ^{1}H NMR (500 MHz) spectrum of 5 in $CDCl_3$

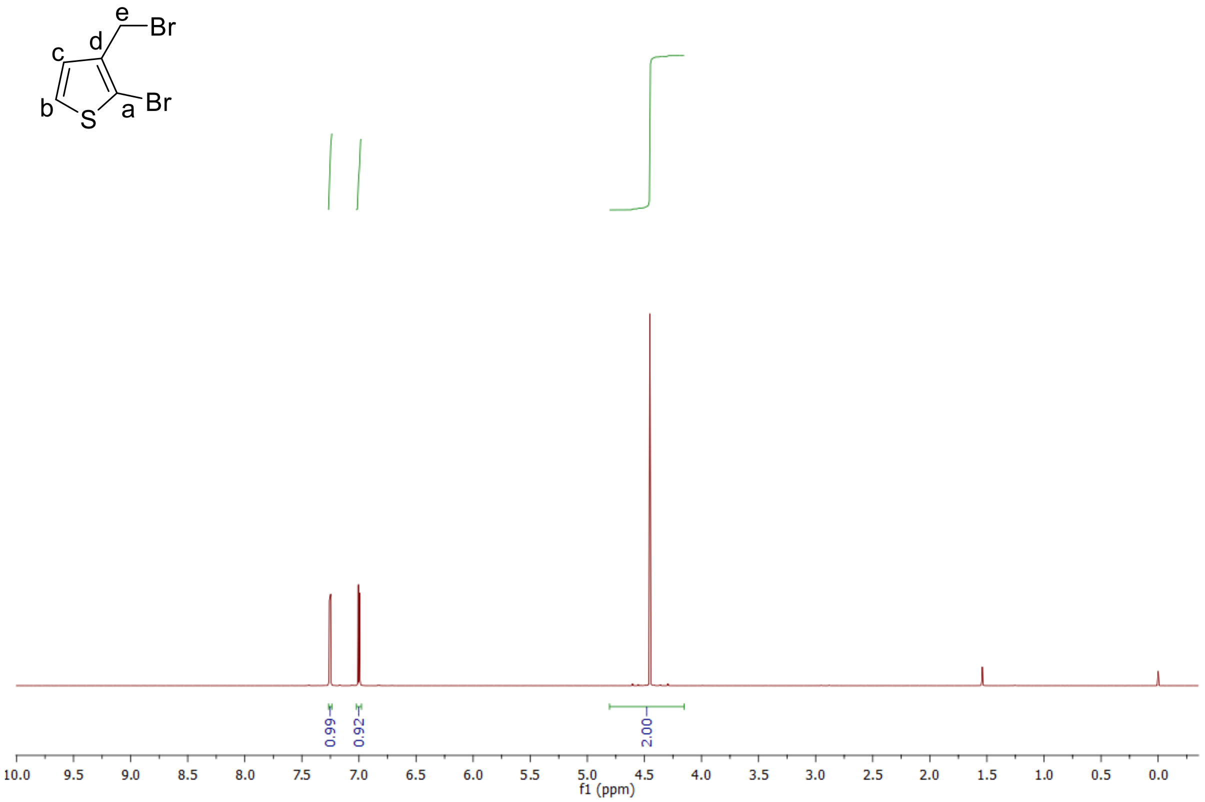

Figure (2) $^{13}C\{^{1}H\}$ NMR (126 MHz) spectrum of 5 in $CDCl_3$

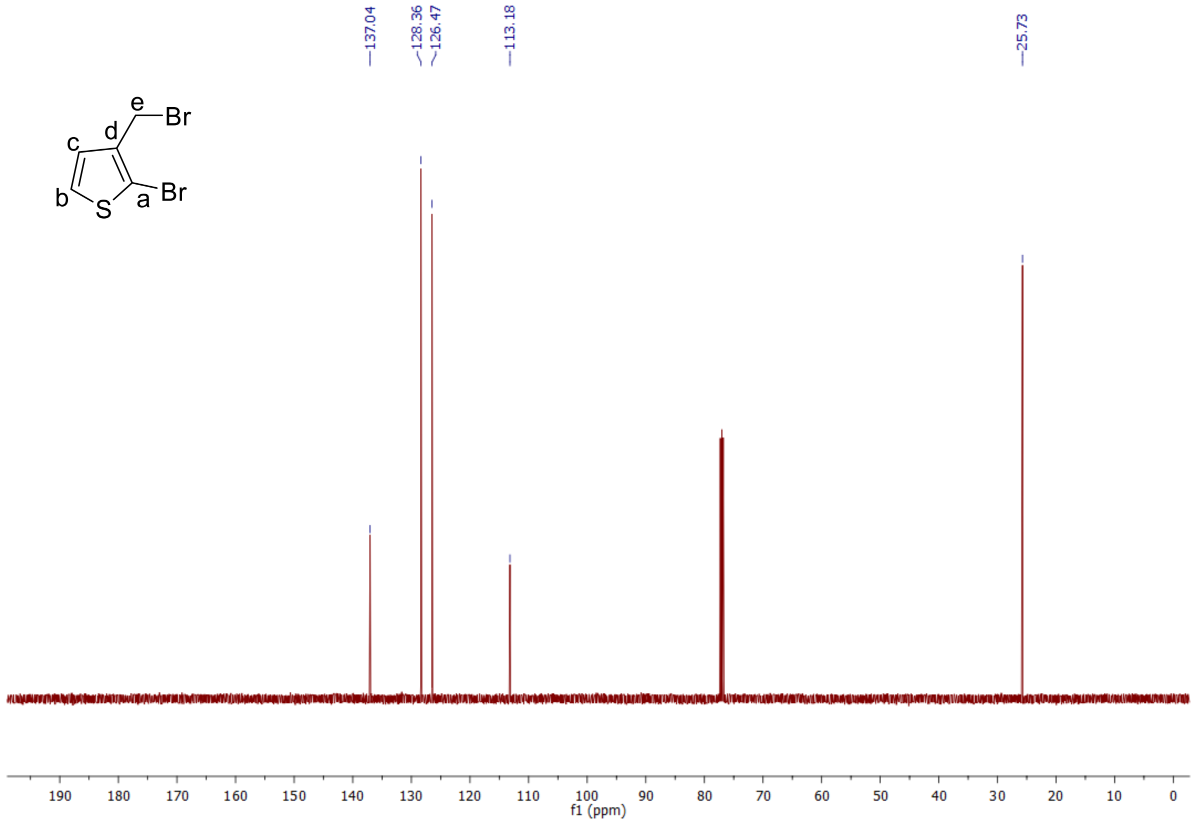

Figure (3) ^{1}H NMR (500 MHz) spectrum of 7 in DMSO-d_6

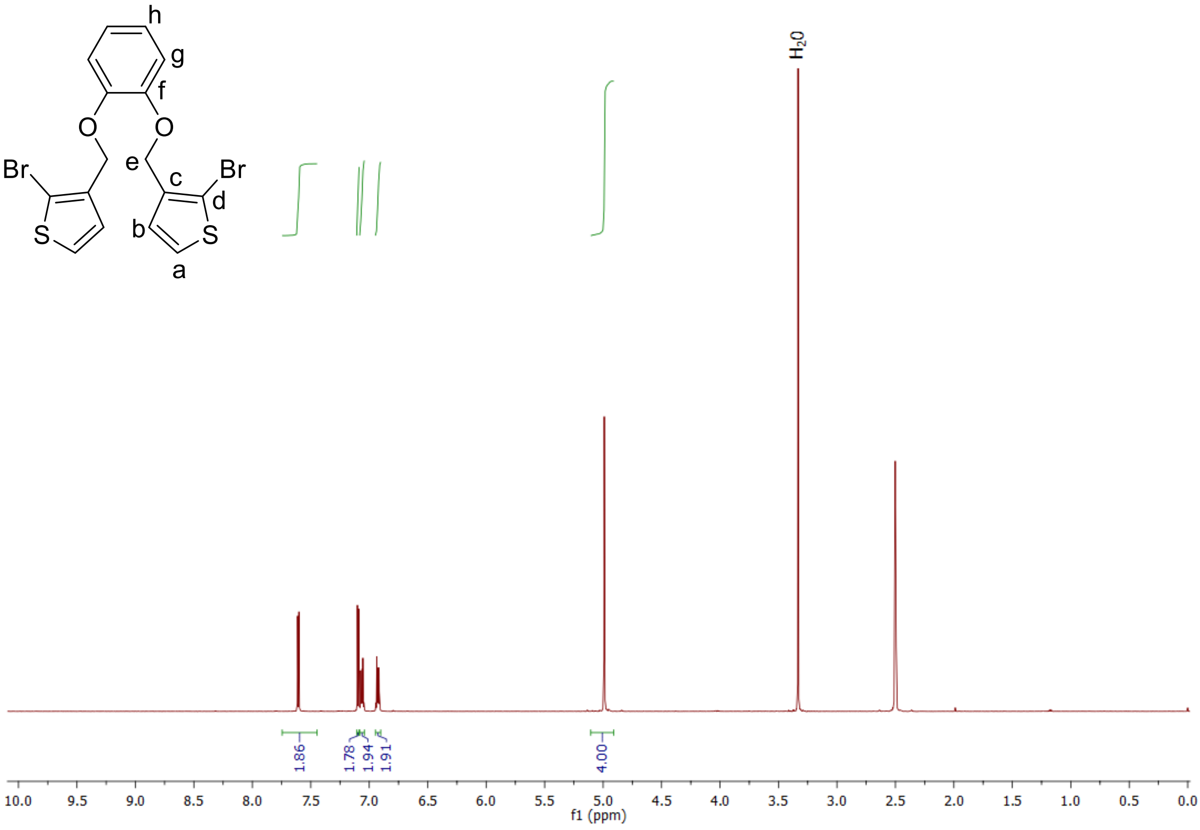

Figure (4) $^{13}C\{^{1}H\}$ NMR (126 MHz) spectrum of 7 in DMSO-d_6

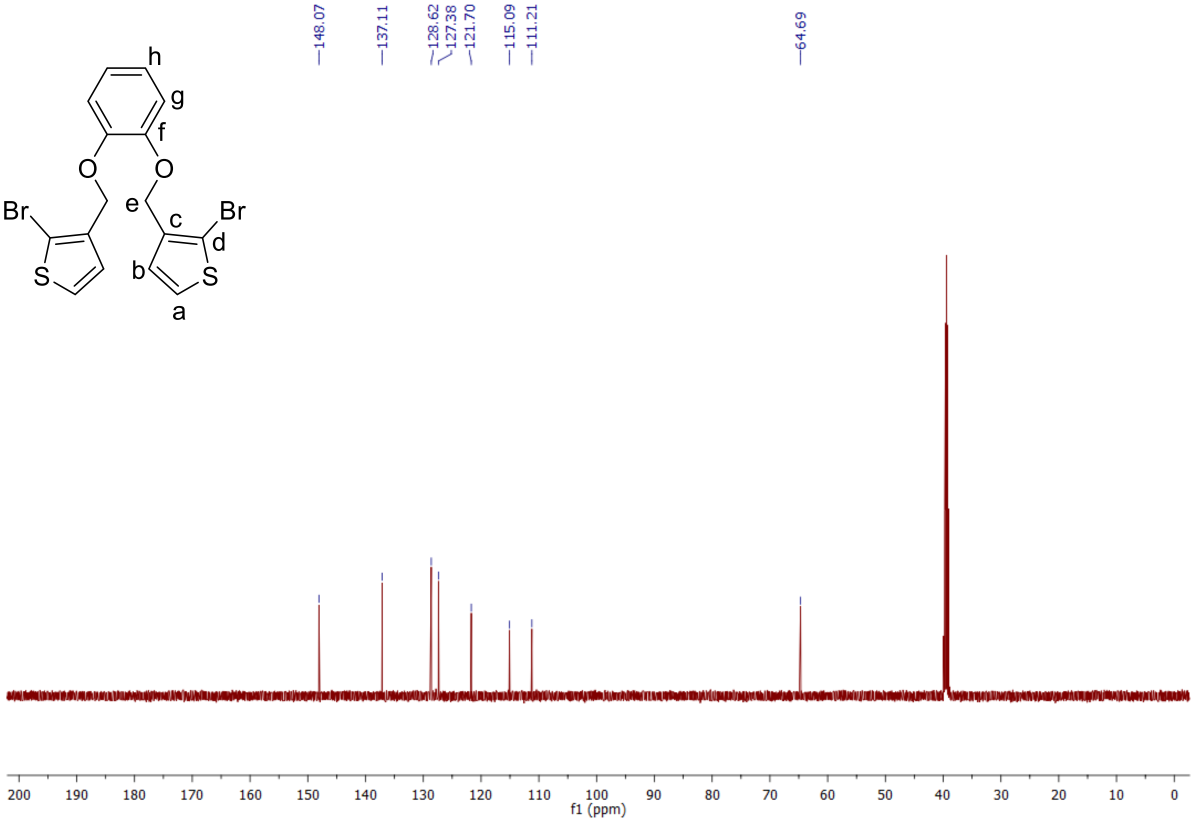

Figure (5) ^{1}H NMR (500 MHz) spectrum of 9a in DMSO-d_6

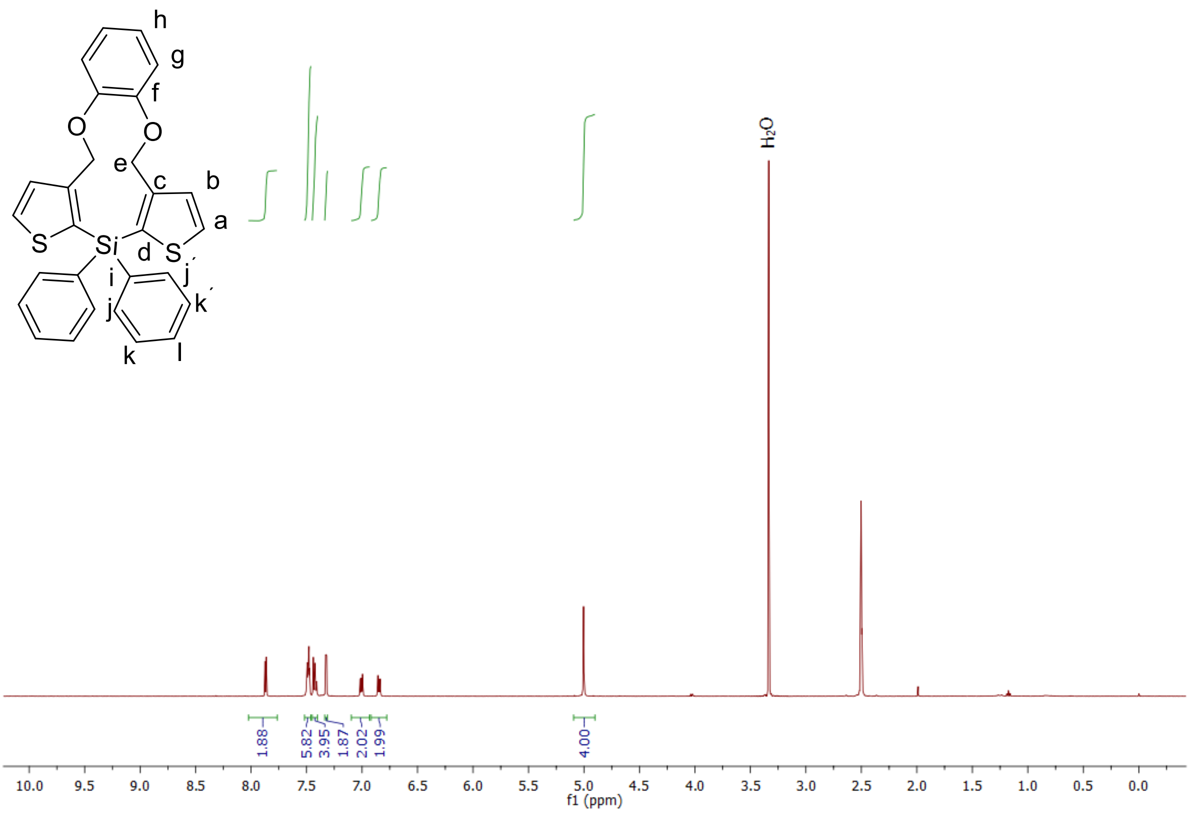

Figure (6) $^{13}C\{^{1}H\}$ NMR (126 MHz) spectrum of 9a in DMSO-d_6

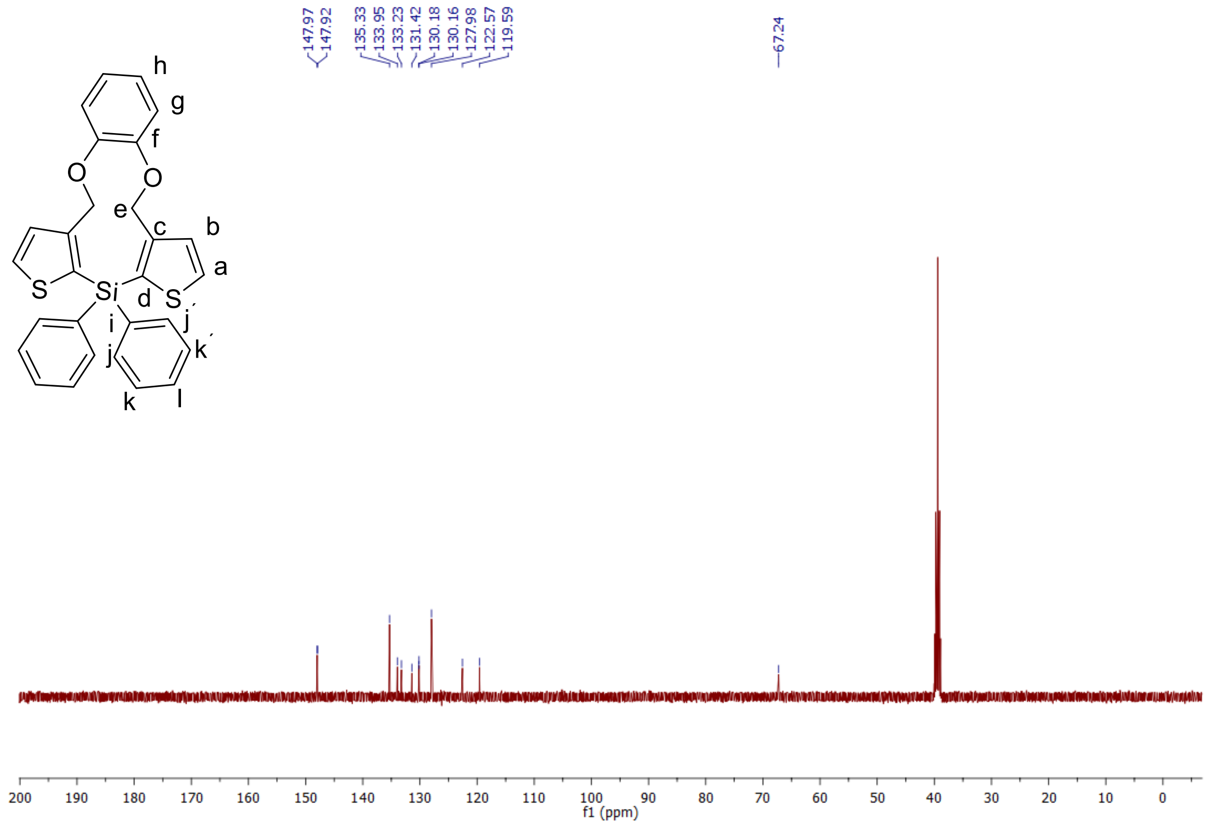

Figure (7) $^{29}Si\{^1H\}$ NMR (119 MHz) spectrum of 9a in DMSO-d_6

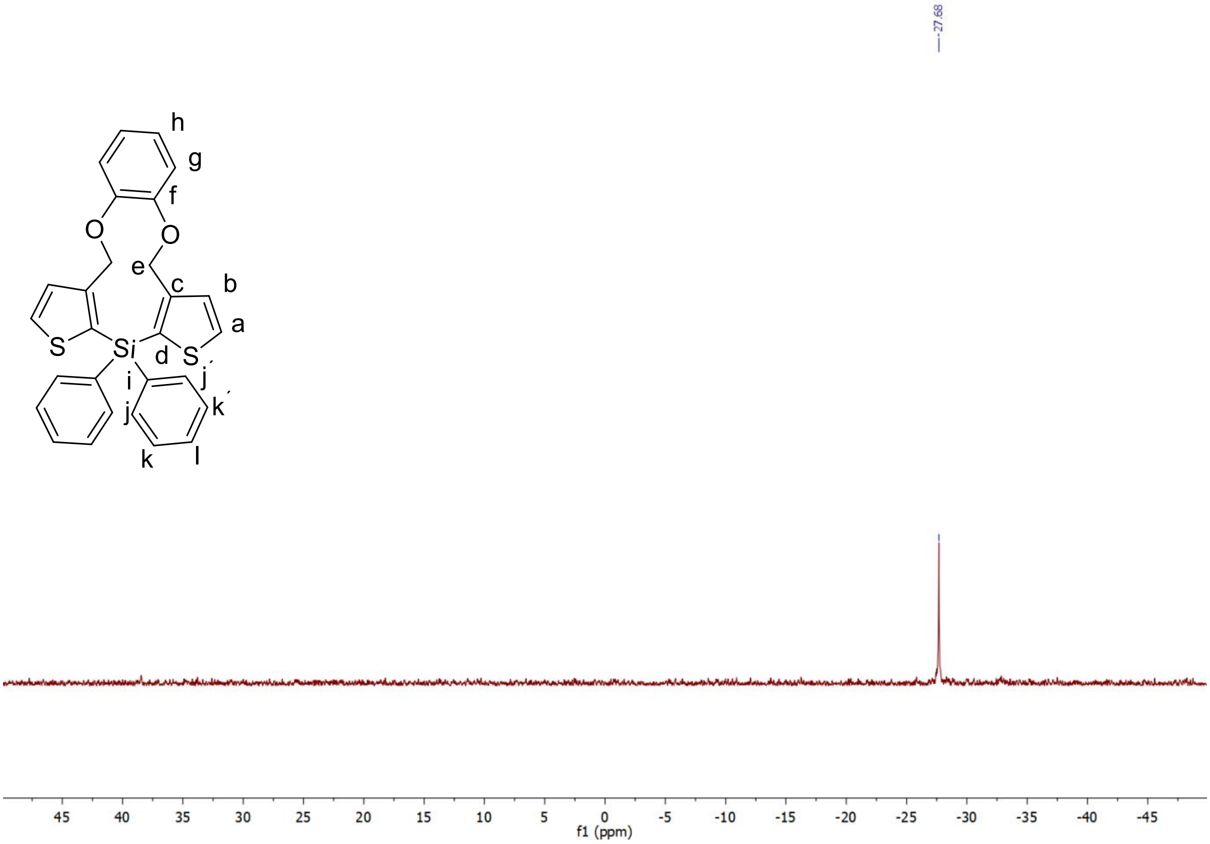

Figure (8) ^1H NMR (500 MHz) spectrum of 9b in DMSO-d_6

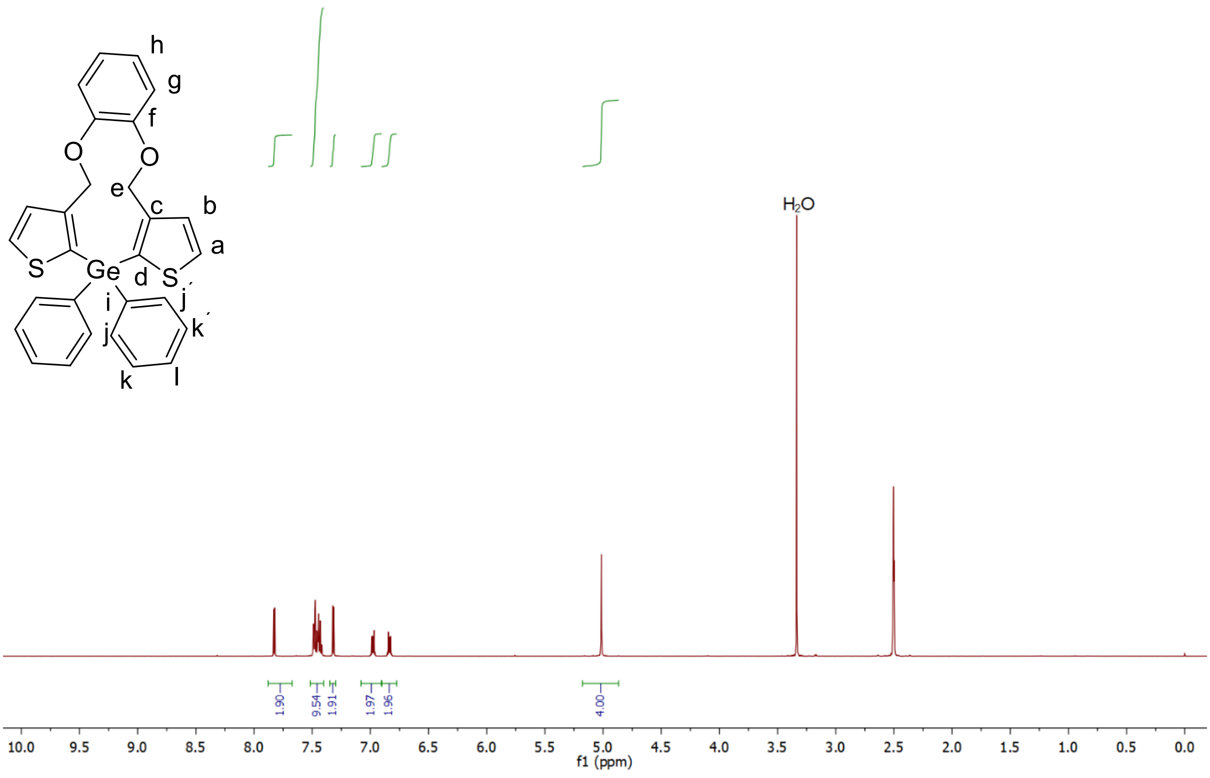

Figure (9) $^{13}C\{^{1}H\}$ NMR (126 MHz) spectrum of 9b in DMSO-d_6

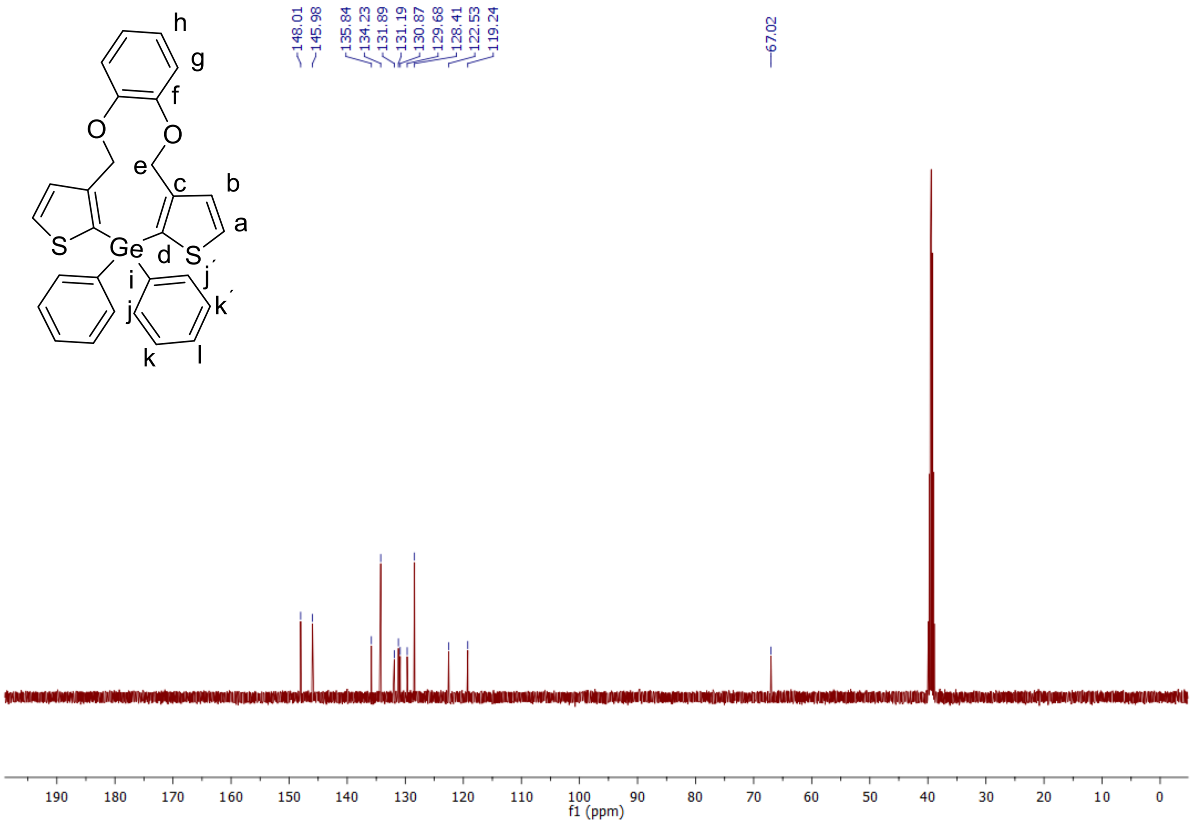

Figure (10) ^{1}H NMR (500 MHz) spectrum of 9c in DMSO-d_6

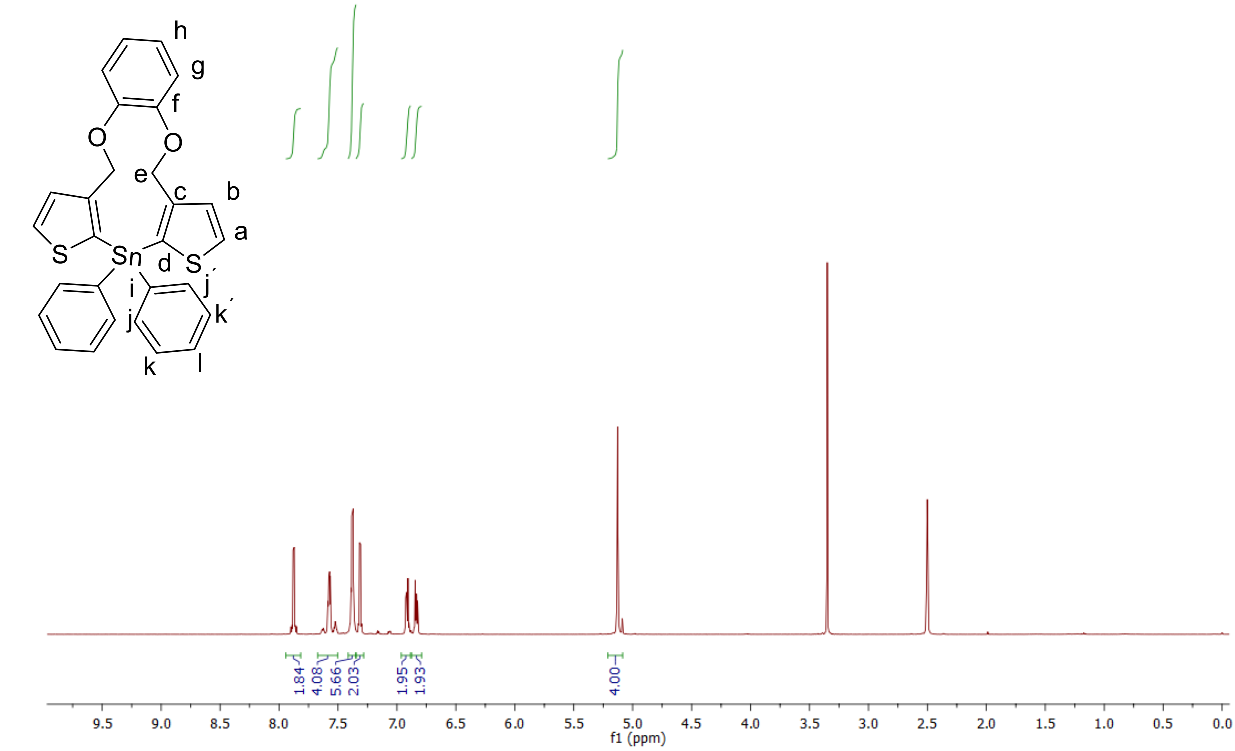

Figure (11) $^{13}C\{^{1}H\}$ NMR (126 MHz) spectrum of 9c in DMSO-d_6

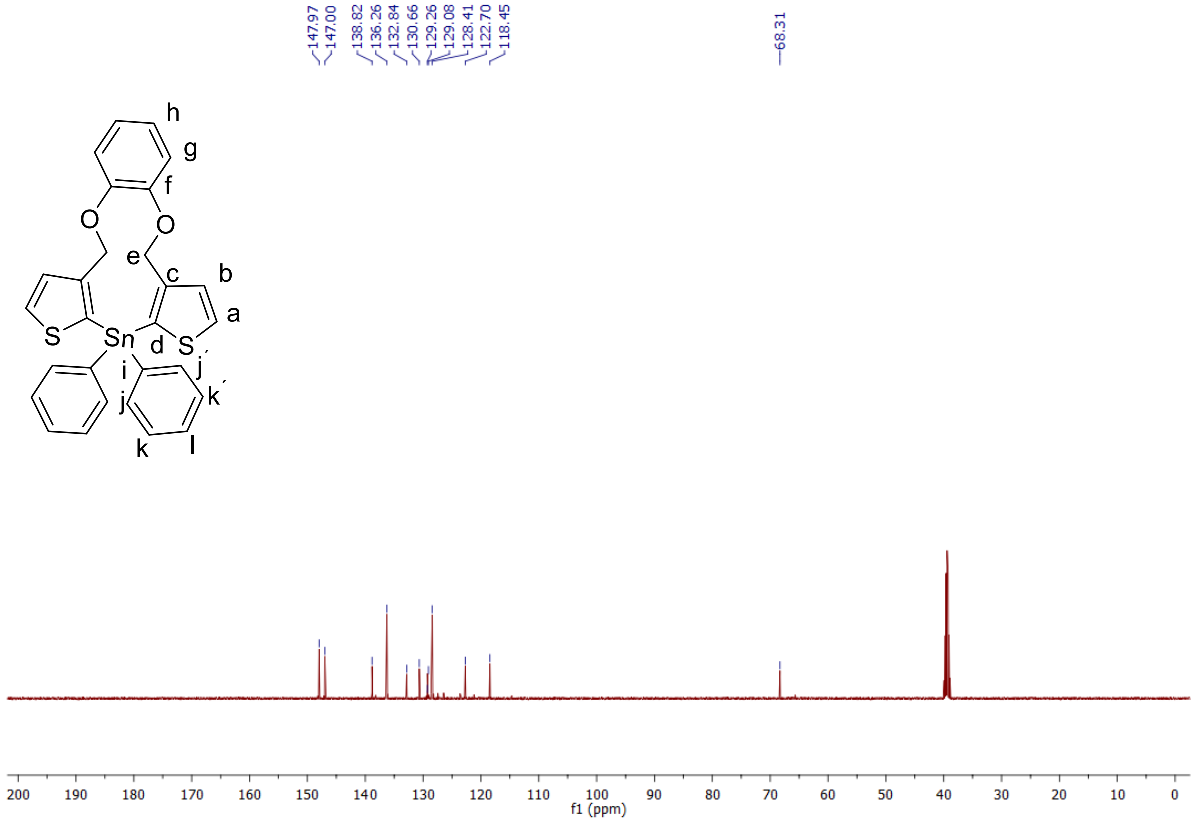

Figure (12) $^{119}Sn\{^{1}H\}$ NMR (187 MHz) spectrum of 9c in DMSO-d_6

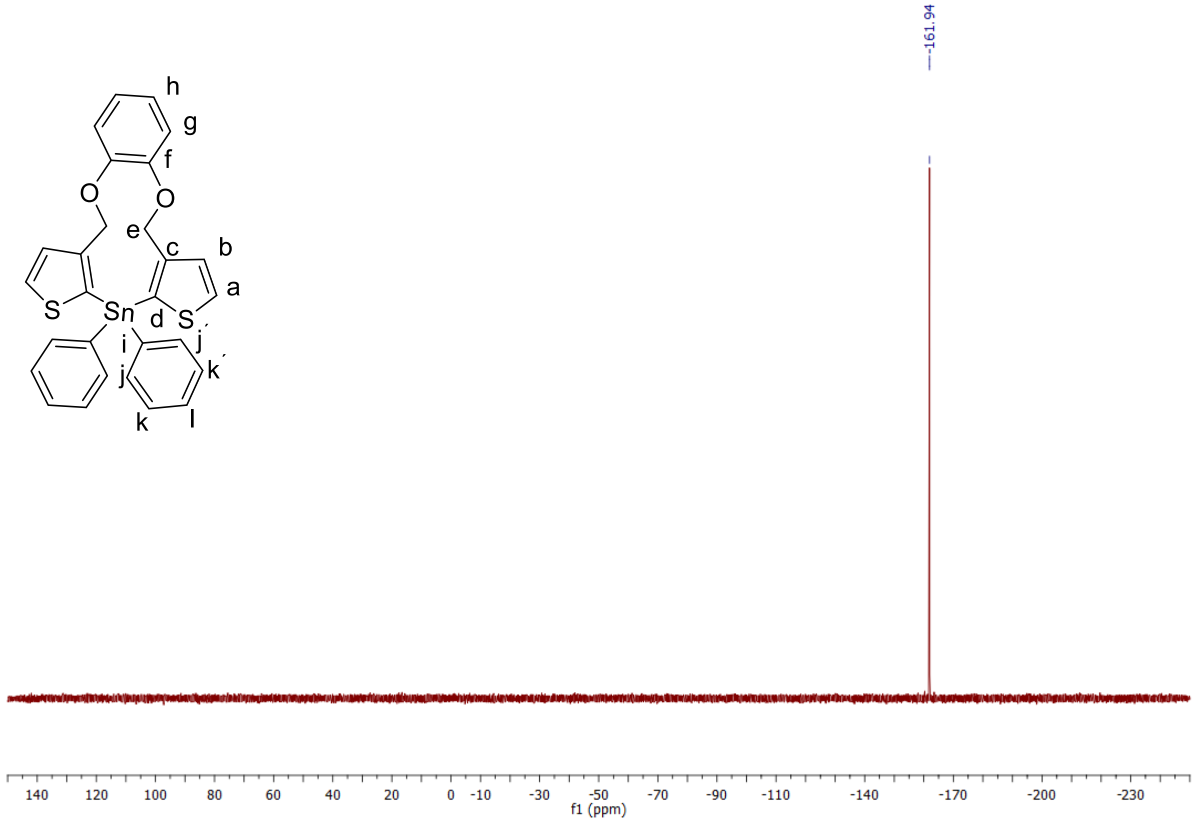

4. Further visualizations of structures 9b and 9c to illustrate the geometrical differences of the conformers

Figure (13) Shape of 9b; H were omitted for clarity.

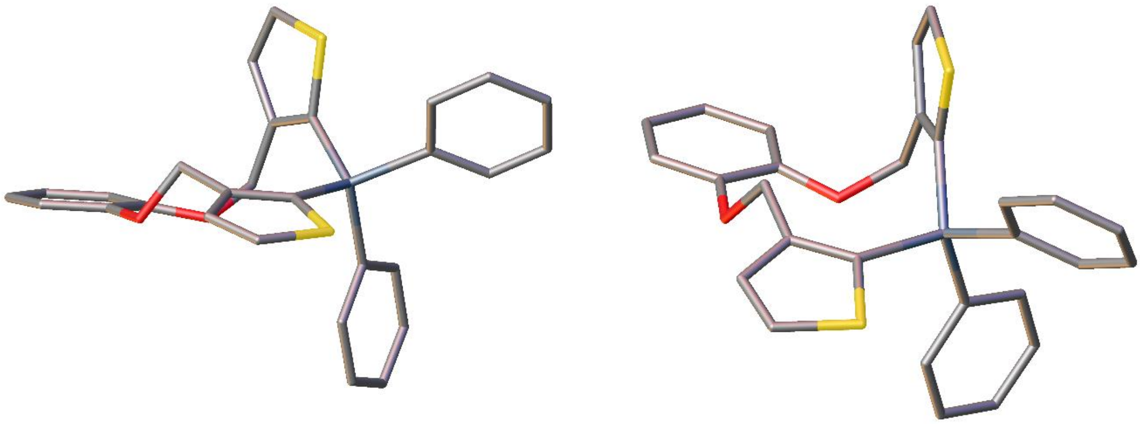

Figure (14) Shape of 9c; H were omitted for clarity.

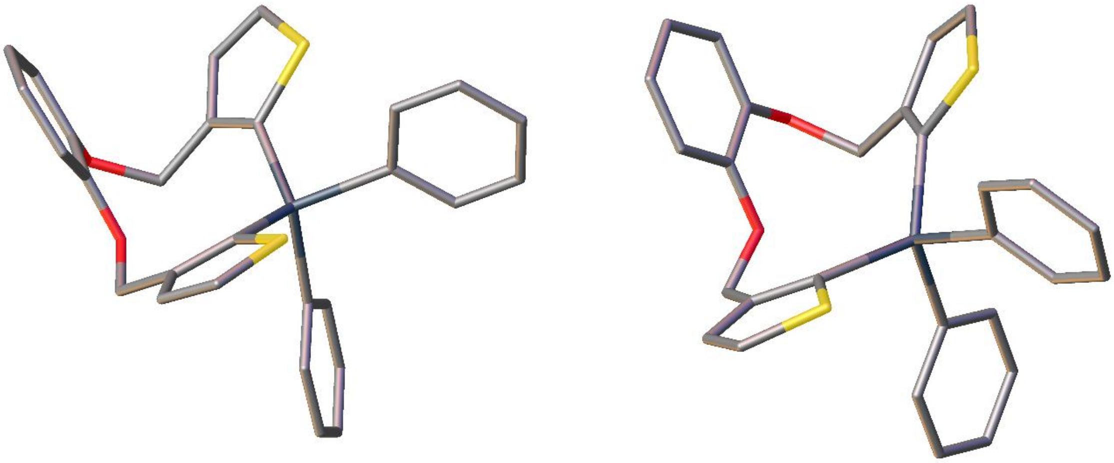

5. Hirshfeld surface analysis

Hirshfeld surface analysis[3] can serve both as a visual aid and as a quantification tool to help understand intermolecular interactions in molecular crystals.[4]

The Hirshfeld surface is the 0.5 isosurface of the weight function between the electron density of a molecule of interest and the electron density of the crystal:

$$w(r) = \sum_m \rho_m(r) \Big/ \sum_c \rho_c(r)$$

Where $\rho_m(r)$ denote the electron densities of all atoms belonging to the molecule of interest and $\rho_c(r)$ the same quantity for the atoms of the crystal. Defined in this way, the Hirshfeld surface fully envelops the molecule of interest. Atoms that are in close vicinity and external to the surface indicate a possible interaction of a neighboring molecule. Similarly, atoms inside and close to the surface may contribute to interactions.

Figure (15) Fingerprint plots for 9b and 9c. External atom distances to the Hirshfeld surface de against internal atom distance d_i

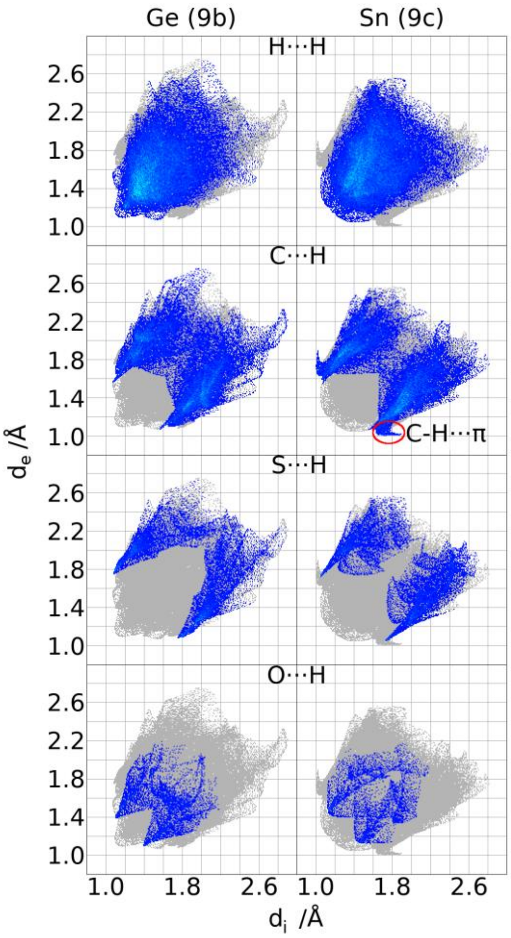

The Hirshfeld surface analysis presented here was performed using the crystal explorer software,[5] employing the Tonto DFT package[6] as the computational backend.

The fingerprint plots in Figure 15 show the closest distance of external H-atoms to the surface (d_e) against the surface-X-distance of a given X-atom (X=H,C,S,O) inside the surface (d_i) for X-H pairs.

From the breakdown of the Hirshfeld surface by nearest X-H distances (Figure 16), it is clear that interactions happen almost exclusively via peripheral hydrogen atoms. At the same time, interactions are similar in both crystal structures, in terms of the surface coverage of the individual X-H contributions.

While crystal packing and intermolecular interactions differ in detail between **9b** and **9c**, both are dominated by H-H and C-H contacts.

Naturally, the percentage of surface area associated with a given interaction also includes long distances that do not contribute significantly to the overall interactions. The fingerprint plots (Figure 15) are more instructive in this regard, as spots closer to the origin correspond to shorter distances, often meaning stronger interactions. Slight differences in C-H, S-H and O-H interactions are visible. Particularly, the edge-face interaction in **9c** (denoted C-H · π) and slightly closer O-H contacts in **9b** are of note.

Figure (16) The relative coverage of the Hirshfeld surface

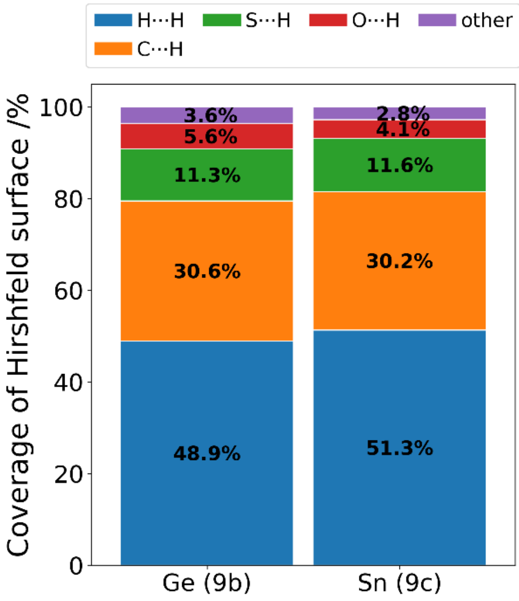

As both molecules are not capable of providing strong electrostatic complementarity, interactions are relatively weak. An impact of the intermolecular interactions to the molecular conformation appears unlikely in light of the high energy differences between conformations as predicted by DFT.

Figure 17 illustrates differences in intermolecular interactions. Near the oxygen lone pairs, the electrostatic potential is negative (indicated by the red Hirshfeld surface). In the case of

9b, the oxygen atoms experiences mainly interactions from one neighboring molecule. In **9c**, this interaction is shared between two adjacent molecules. This example is not meant to suggest the importance of this particular interaction, but instead to show that the differences are numerous and not only related to any one particular effect.

Figure (17) The Hirschfeld surface of 9b (left) and 9c (right) colour-coded for electrostatic potential. Red for negative and blue for positive.

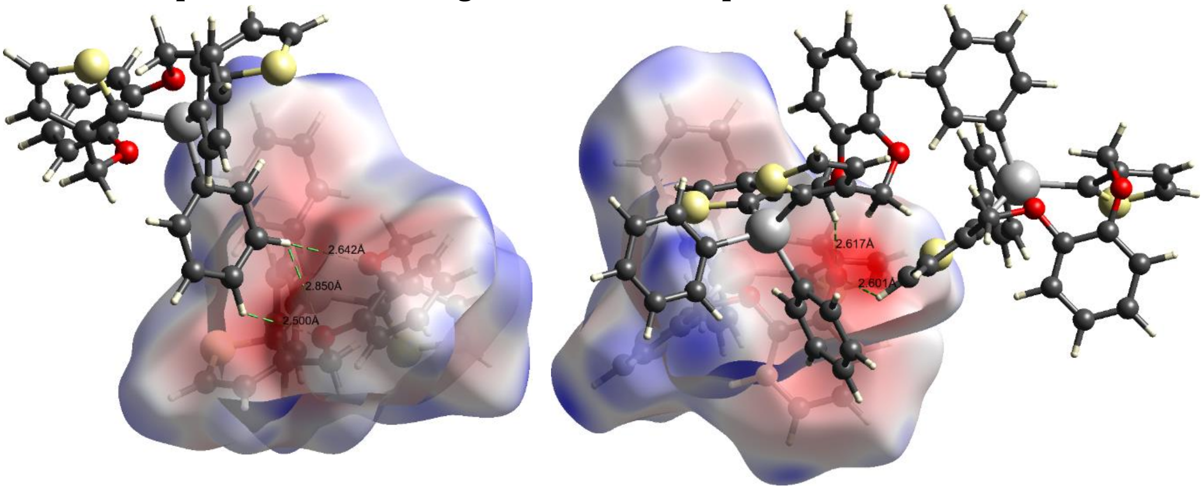

6. Summary of the crystal data for compounds 9b and 9c

Table 3 Crystal data and structure refinement for compound 9b.

Empirical formula	$C_{28}H_{22}O_2S_2Ge$
Formula weight	527.16
Temperature/K	83(2)
Crystal system	Triclinic
Space group	P-1
a/Å	9.2384(5)
b/Å	10.8384(6)
c/Å	12.5164(6)
α/°	78.460(2)
β/°	70.472(2)
γ/°	86.742(3)
Volume/Å^3	1157.23(11)
Z	2
ρ_{calc}g/cm^3	1.513
μ/mm^{-1}	1.53
F(000)	540.0
Crystal size/mm^3	0.327 × 0.189 × 0.169
Reflections collected	42146

Independent reflections	7119 [R_{int} = 0.0292, R_{sigma} = 0.0217]
Data/restraints/parameters	7119/0/298
Goodness-of-fit on F^2	1.023
Final R indexes [I>=2σ (I)]	R_1 = 0.0235, wR_2 = 0.0558
Final R indexes [all data]	R_1 = 0.0299, wR_2 = 0.0584
Largest diff. peak/hole / e $Å^{-3}$	0.45//-0.37

Table 2 Crystal data and structure refinement for Compound 9c.

Empirical formula	$C_{28}H_{22}O_2S_2Sn$
Formula weight	573.26
Temperature/K	100.0
Crystal system	monoclinic
Space group	$P2_1/n$
a/Å	9.2379(4)
b/Å	10.0757(4)
c/Å	25.7667(10)
α/°	90
β/°	98.5940(10)
γ/°	90
Volume/$Å^3$	2371.39(17)
Z	4
ρ_{calc}g/cm^3	1.606
μ/mm^{-1}	1.278
F(000)	1152.0
Crystal size/mm^3	0.35 × 0.22 × 0.2
Reflections collected	49280
Independent reflections	6312 [R_{int} = 0.0598, R_{sigma} = 0.0332]
Data/restraints/parameters	6312/0/299
Goodness-of-fit on F^2	1.099
Final R indexes [I>=2σ (I)]	R_1 = 0.0268, wR_2 = 0.0553
Final R indexes [all data]	R_1 = 0.0336, wR_2 = 0.0575
Largest diff. peak/hole / e $Å^{-3}$	0.65/-0.75

7. Bibliography

1. Sheldrick, G., A short history of SHELX. *Acta Crystallographica Section A* **2008,** 64, (1), 112-122.
2. Nitti, A.; Signorile, M.; Boiocchi, M.; Bianchi, G.; Po, R.; Pasini, D., Conjugated Thiophene-Fused Isatin Dyes through Intramolecular Direct Arylation. *The Journal of Organic Chemistry* **2016,** 81, (22), 11035-11042.
3. McKinnon, J. J.; Mitchell, A. S.; Spackman, M. A., Hirshfeld Surfaces: A New Tool for Visualising and Exploring Molecular Crystals. *Chemistry – A European Journal* **1998,** 4, (11), 2136-2141.
4. Spackman, M. A.; Jayatilaka, D., Hirshfeld surface analysis. *CrystEngComm* **2009,** 11, (1), 19-32.
5. Turner, M. J.; McKinnon, J. J.; Wolff, S. K.; Grimwood, D. J.; Spackman, P. R.; Jayatilaka, D.; Spackman, M. A., CrystalExplorer17. *University of Western Australia. http://hirshfeldsurface.net.* **2017,**.
6. Jayatilaka, D.; Grimwood, D. J. In *Tonto: A Fortran Based Object-Oriented System for Quantum Chemistry and Crystallography*, Computational Science — ICCS 2003, Berlin, Heidelberg, 2003//, 2003; Sloot, P. M. A.; Abramson, D.; Bogdanov, A. V.; Gorbachev, Y. E.; Dongarra, J. J.; Zomaya, A. Y., Eds. Springer Berlin Heidelberg: Berlin, Heidelberg, pp 142-151.
